# Genomic evolution of the class *Acidithiobacillia*: deep-branching Proteobacteria living in extreme acidic conditions

**DOI:** 10.1038/s41396-021-00995-x

**Published:** 2021-05-18

**Authors:** Ana Moya-Beltrán, Simón Beard, Camila Rojas-Villalobos, Francisco Issotta, Yasna Gallardo, Ricardo Ulloa, Alejandra Giaveno, Mauro Degli Esposti, D. Barrie Johnson, Raquel Quatrini

**Affiliations:** 1 Fundación Ciencia & Vida, Santiago, Chile; 2 Facultad de Ciencias de la Vida, Universidad Andres Bello, Santiago, Chile; 3 ANID–Millennium Science Initiative Program–Millennium Nucleus in the Biology of the Intestinal Microbiota, Santiago, Chile; 4 Facultad de Medicina y Ciencia, Universidad San Sebastián, Providencia, Santiago, Chile; 5 PROBIEN (CCT Patagonia Confluencia-CONICET, UNCo), Departamento de Química, Facultad de Ingeniería, Universidad Nacional del Comahue, Neuquén, Argentina; 6 Center for Genomic Sciences, Universidad Nacional Autónoma de México (UNAM), Cuernavaca, México; 7 School of Biological Sciences, Bangor University, Bangor LL57 2UW, UK, and Faculty of Health and Life Sciences, Coventry University, Coventry, UK

**Keywords:** Bacterial evolution, Bacterial genomics, Phylogenetics

## Abstract

Members of the genus *Acidithiobacillus*, now ranked within the class *Acidithiobacillia*, are model bacteria for the study of chemolithotrophic energy conversion under extreme conditions. Knowledge of the genomic and taxonomic diversity of *Acidithiobacillia* is still limited. Here, we present a systematic analysis of nearly 100 genomes from the class sampled from a wide range of habitats. Some of these genomes are new and others have been reclassified on the basis of advanced genomic analysis, thus defining 19 *Acidithiobacillia* lineages ranking at different taxonomic levels. This work provides the most comprehensive classification and pangenomic analysis of this deep-branching class of Proteobacteria to date. The phylogenomic framework obtained illuminates not only the evolutionary past of this lineage, but also the molecular evolution of relevant aerobic respiratory proteins, namely the cytochrome *bo*_3_ ubiquinol oxidases.

## Introduction

Members of the genus *Acidithiobacillus* are among the most widely studied extremely acidophilic prokaryotes [1]. The genus comprises Gram-negative autotrophic bacteria that are obligate acidophiles. While all *Acidithiobacillus* spp. can catalyze the dissimilatory oxidation of sulfur compounds, some members of the genus grow by oxidizing ferrous iron, using oxygen as electron acceptor [2]. These combined physiological traits are used extensively in biotechnological applications, including the biomining of metal sulfide ores [3]. From a fundamental perspective, the acidithiobacilli are model bacteria for the study of chemolithotrophic energy conversion reactions and pathways under acidic conditions (reviewed in ref. [4]). More recently, they have provided key insights into the evolution of aerobic respiration [5].

First considered to be members of the *Betaproteobacteria* [6], the acidithiobacilli were later assigned to *Gammaproteobacteria* [7]. With the advance of genome sequencing and phylogenomic analysis, they were subsequently repositioned as a sister class to the *Beta*-, *Gamma*-, and *Epsilonproteobacteria* [8], together with *Thermithiobacillus tepidarius* [9]. The class *Acidithiobacillia* (often referred to as the acidithiobacilli) contains a single order and two families, the *Acidithiobacillaceae* and the *Thermithiobacillaceae*, the latter including non-acidophilic taxa. *T. tepidarius* and *Thermithiobacillus plumbiphilus* are the only acknowledged species of the *Thermithiobacillaceae* [10]. Like *Acidithiobacillus* spp., these bacteria are aerobic, obligately chemolithoautotrophic sulfur-oxidizers, and in common with only one *Acidithiobacillus* sp. (*Acidithiobacillus caldus*), moderately thermophilic [10]. Considering the high taxonomic rank of the *Acidithiobacillia*, little attention has been paid to their true diversity so far, thereby leaving a considerable gap in our knowledge of these extremophiles.

Variability among members of this class is evident. Phenotypically, the acidithiobacilli span a wide range of growth temperatures, ranging from >50 °C in *T. tepidarius* [11] and *A. caldus* [12] to 4 °C in *Acidithiobacillus ferrivorans* [13], and of pH optima, ranging from 7 in *T. tepidarius* [11] to 1.8 in *Acidithiobacillus ferrianus* [14], with *Acidithiobacillus sulfuriphilus* growing in a pH range of 5 units [15]. While knowledge of the physiological and phylogenetic diversity of *Thermithiobacillus* is currently limited, bacteria confirmed as members of the *Acidithiobacillus* genus have been isolated from many different settings since the 1950s (reviewed in ref. [16]). Many isolates [17] and clones [18] have been assigned to distinct phylogenetic subgroups or genomovars [19], some of which are probably novel species [20]. Current consensus recognizes ten species within the *Acidithiobacillus* genus, five of which are able to couple the dissimilatory oxidation of elemental sulfur and reduced inorganic sulfur compounds (but not ferrous iron) to molecular oxygen: *Acidithiobacillus thiooxidans* [21], *Acidithiobacillus albertensis* [22], *A. caldus* [12], and *Acidithiobacillus sulfuriphilus* [15]. The other species are additionally capable of using ferrous iron as an electron donor (in the presence of oxygen) or ferric iron as an electron acceptor, coupled to sulfur oxidation (under anaerobic conditions): *Acidithiobacillus ferrooxidans* [23], *A. ferrivorans* [13], *Acidithiobacillus ferridurans* [24], *Acidithiobacillus ferriphilus* [25], and *A. ferrianus* [14]. The ability to grow with hydrogen as electron donor using either oxygen or ferric iron as electron acceptor is also widespread among the acidithiobacilli [26]. While some members of the *Acidithiobacillus* genus are well characterized physiologically, many isolates remain unassigned or uncultured; therefore, their metabolic characteristics remain unknown [27]. As more genomes and metagenome-assembled genomes (MAGs) assigned to acidithiobacilli are sequenced, the knowledge gap between genomic information and physiology has expanded [28, 29].

Nonetheless, accumulating evidence confirms that most species of the *Acidithiobacillus* genus are highly diverse at the genomic level, exhibiting both species-to-species and strain-to-strain heterogeneity [30]. Intraspecific variations can be accounted for by diverse plasmids present [31], and by a highly diverse set of integrated mobile genetic elements [32–34]. Recently, pangenomic studies have provided additional insights into the core and exclusive functions of certain species of *Acidithiobacillus* [35, 36]. However, studies performed so far have (i) overlooked the existence of different taxonomic ranks within the class *Acidithiobacillia*, (ii) involved few representative strains of each species, and/or (iii) undertaken biased sampling efforts, recovering strains from a limited range of habitats or geographical origins.

Recognizing the limits of current knowledge of *Acidithiobacillia*, we elected to test the hypothesis that the *Acidithiobacillia* class encompasses a greater number of genera and species than previously acknowledged. To this end, we undertook systematic efforts to sample, culture, and sequence new representatives of the class from diverse environments. These included unconventional and little-reported niches, such as geothermal acidic watersheds and near-neutral mineral environments. In addition, we have sequenced the missing genomes of type strains of validated species of acidithiobacilli. This work has aimed to reconstruct a robust genome-based phylogeny enabling the exploration of the ecological and metabolic origin of acidithiobacilli on the basis of their extant representatives. Our work represents the state of the art for the knowledge of these extremely acidophilic bacteria and their physiology, and sheds light into their evolutionary history.

## Materials and methods

### Bacterial strains and growth conditions

Bacterial strains used in this study are listed in Table S1a. These were obtained at the DSMZ (https://www.dsmz.de) or ATCC (https://www.atcc.org) culture collections or sourced from the acidophile culture collections maintained at Bangor University (Wales, UK) and at Fundación Ciencia y Vida (Santiago, Chile). All available strains were classified in phylogenetic groups utilizing the 16S rRNA gene sequence and oligotyped as defined in Nuñez and colleagues [20]. Strains were grown in liquid culture under aerobic conditions (200 r.p.m.), using mineral salts medium with trace elements [37] supplemented with either ferrous iron (20–50 mM FeSO_4_) or tetrathionate (5 mM K_2_O_6_S_4_) as energy sources. Temperature incubation conditions and pH adjustment followed recommended optima for each species (Table S2).

### DNA isolation, library construction, and sequencing

Stationary phase cultures used for nucleic acid purification were centrifuged at 8000 × *g* for 10 min and harvested cells were stored as pellets (0.5 g wet weight) at −80 °C until DNA isolation. Total DNA isolation was performed as in Nieto and colleagues [38]. DNA was resuspended in 100 µl TE buffer (Tris 10 mM, EDTA 1 mM, pH 8) and tested for integrity and purity using gel electrophoresis and spectrophotometry as standard quality check procedures. DNA concentrations were quantified with PicoGreen and a Synergy H1 microplate reader using a Take3™ Micro-Volume Plate (BioTek Instruments, Inc.), and diluted in ultrapure water at concentrations suitable for sequencing workflows (>30 ng/µl). Sequencing library preparation and DNA sequencing were conducted at CD Genomics (https://www.cd-genomics.com) NY, USA. DNA was sequenced using Illumina sequencing technology on the HiSeq 2000 platform, producing 150 bp paired reads with a targeted insert size of 500 bp. To ensure robust assemblies sequencing runs yielding >1,500,000 paired-end reads per sample were further processed.

### Genome assembly and recovery from databases

Prior to analysis, the raw sequencing reads were preprocessed using Trimmomatic v0.36 [39] to trimm adaptors and to remove low quality reads. Read quality was verified by FastQC analysis (http://www.bioinformatics.babraham.ac.uk/projects/fastqc/). Reads with a quality score > Q30 were retained and assembled *de novo* using Velvet v1.2.10 [40] and *k*-mer lengths in the 21–199 range. Genome drafts obtained were checked for contamination and completeness as in Raes et al. [41] and uploaded to the WGS NCBI Genome database. Resulting assembly statistics are summarized in Table S1b, along with sequence deposit information. Additional public genomes of *Acidithiobacillus* spp. and *Thermithiobacillus* spp. were obtained from NCBI (https://www.ncbi.nlm.nih.gov/assembly/) as of March 2020 (Table S1b), except for the genome of strain DSM 16786, which was obtained online (http://biominingdb.cmm.uchile.cl/genomes/At_ferrooxidans_Wenelen/) and uploaded to NCBI with authorization of Dr. Patricio Martinez Bellange (ex. CodelcoTech, Santiago, Chile) for data normalization.

### Gene-calling and annotation

Gene-calling and annotation were performed using the NCBI Prokaryotic Genome Annotation Pipeline (PGAP [42, 43]). Two genome sequences of low quality (JNNH01 and DDOU01) were annotated through the RAST pipeline (Rapid Annotation using Subsystem Technology [44]). Recovered annotations were validated against curated protein profiles stored in TIGRFAM and PRK, using Hidden Markov models. Identification of specific domains in selected proteins was done with RPS-BLAST v2.2.26 versus CDD database [45]. Elimination of overlapping motifs and redundant results were done with rpsbproc v0.11. All predicted proteins were also analyzed with additional resources, including the KO profiles KEGG and COG databases as of July 2020, using SqueezeMeta [46].

### OGRIs calculations

Overall genome relatedness indexes (OGRIs) were derived from all possible pairwise genome comparisons, using the following tools. The average nucleotide identities based either on Blast (ANIb) or Mummer (ANIm) as alignment algorithms and tetranucleotide frequencies (TETRA) were calculated using a Python module implemented by Pritchard et al. [47] available at https://github.com/widdowquinn/pyani. The *in silico* digital DNA–DNA hybridization index (dDDH) was assessed using the Genome-to-Genome Distance Calculator with recommended formula 2 [48], and species cutoff limits defined by Meier-Kolthoff and colleagues [49] available at http://ggdc.dsmz.de. Tetranucleotide-derived *Z*-value Manhattan Distance (TZMD) was calculated based on TETRA values, using TZMD v1.0 [50] (https://github.com/Yizhuangzhou/TZMD). The average amino acid identity (AAI) was calculated, using the CompareM program (https://github.com/dparks1134/CompareM) and the aai.rb implementation from the Kostas Lab [51], downloaded from github (https://github.com/lmrodriguezr/enveomics; commit signature: fae592f) and run using default parameters. Comprehensive OGRI matrices resulting from these analyses are presented in Tables S3 and S4.

### Validation of novel lineages

Sequences assigned to novel *Acidithiobacillia* class species represented by a single genome representative were searched against information available in public nucleotide sequence databases (genes, nr and env_nr, SRA) as of August 2020, using either 16S rRNA gene sequences or selected class core genes (e.g., selected ribosomal or conserved proteins, CPs). Fragment recruitment alignments of reads from the SRA were done with Bowtie2 V2.2.5 [52]. Recovered 16S rRNA gene sequences were oligotyped in Nuñez and colleagues [20]. Metadata extracted from each sequence deposit was also recovered and summarized in Table S1.

### Comparative genomics methods

The predicted amino acid sequences of all open reading frames identified in the genomes of interest were analyzed in search for orthology. Genome comparisons were performed using the GET_HOMOLOGUES [53] software package v3.3.2. Orthology was determined using COGtriangles (v2.1) as clustering algorithm and triangle reciprocal hits were clustered in Protein Families (PFs). BLAST pairwise alignment cutoffs were set at 75% coverage, *E*-values at 10E-5 and other parameters were used as default. The number of copies per ortholog and per genome was scored using in-house Perl/Phyton scripts. Identity between multi-copy genes was assessed to discriminate orthologs form paralogs (*E*-value cutoff = 10E-5; identity cutoff = 60%), and results were curated on the basis of genomic context analysis. Pangenome metrics, including the size of core, flexible, and pangenome were calculated as in Tettelin and colleagues [54]. Phyletic patterns were constructed by scoring the presence/absence of representatives of each PF in each genome using parse_pangenome_matrix.pl and other in-house scripts. Data analysis and visualization were conducted using the R package tidyverse v1.3.0. Gene vicinity information was computed and gene contexts of interest recovered using in-house scripts. Concurrence per vicinity and per genome was scored and used to construct gene association networks using Cytoscape 3.6.1 [55], where nodes are the variants and the edges are the presence/absence data or the statistical correlation data.

### Phylogenomic reconstruction and analysis

Different sets of ribosomal proteins (RPs; Table S5), conserved core proteins (Table S6), or protein families of ecophysiological/evolutionary interest (Table S7) were aligned using the MAFFT v7.310 software with the L-INS-I method [56]. Protein alignments were generated for each individual protein family using MAFFT L-INS-i (settings maxiterate 1000 and localpair). All alignments were trimmed using trimAl with gap threshold 0.5. The resulting alignments were checked and further refined manually. Phylogenetic trees were generated by three methods. First, maximum likelihood (ML) trees were reconstructed using PhyML v3.0 [57]. The topology of the tree and the length of the branches were optimized using SMS v1.8.4. The phylogenetic tree was assessed using 1000 bootstrap replicates. A second tree was generated using Bayesian analysis with MrBayes v3.2.6 [58] and/or the BEAST 2.6.2 package [59]. Manually curated alignments were first prepared as xml files containing the detailed settings for the phylogenetic analysis using BEAUti 2 app [59]. Routinely, the settings to run the BEAST analysis were four gamma categories with shape 0.3 (or other empirically estimated values); the WAG substitution model; Relaxed Clock Log model and the gamma option for the Yule birth model (other priors were left in their default setting); ~2 million iterations for the Markov chain of Monte Carlo analysis with five pre-burnin. Trees were stored every 1000 iterations and then usually reduced by 10% burnin to generate Maximal Clade Credibility (MCC) output files with minimal posterior value of 0.1. MCC trees were graphically elaborated with the FigTree program, and sometimes visualized using the program DensiTree 2 [59]. With Mr. Bayes, the analysis was run for 300,000 generations, and trees were saved every 100 generations. Posterior probabilities were calculated after discarding the first 30% of trees [58] with a nucmodel and MCMC (Markov chain Monte Carlo). A third tree inference was undertaken using FastMe v2.0 [60] to generate neighbor-joining (NJ) trees; model substitution was WAG for amino acids, and bootstrap support was set at 1000 replicates.

### Data visualization and manipulation

FigTree v1.4.3 was used for tree visualization and manipulation (http://tree.bio.ed.ac.uk/software/figtree/). Summary statistics and figures were computed using R packages: gdata v2.18.0, dplyr v1.0.2, plotly v4.9.0, ggplot2 v3.2.1, scales v1.0, RColorBrewer v1.1.2, readr v1.2.1, on Rbase v3.6.1 implemented in Rstudio v1.2.50001 [61]. Improvement of vectorial figures was made using Inkscape v1.0 (https://inkscape.org).

## Results

### Genomic diversity within the *Acidithiobacillia*

We undertook a systematic study to analyze nearly 100 *Acidithiobacillus* genomes recovered from strains originating at geographically widespread locations and environmentally diverse habitats, half of which were added in this study (Table S1). This collection spans cultured representatives of all validated species of the taxon and candidate lineages and sublineages.

Genome size, protein-coding sequences, and G + C mol% differences within and between lineages (defined by 16S rRNA gene phylogenetic analysis and oligotyping) indicated that significant genetic variations occur within the *Acidithiobacillia* class (Table S8). A clear example of intraclade variation is *A. thiooxidans*, the strain genomes of which span at least six 16S rRNA gene sequence oligotypes and have about one-third difference in gene content. Data collected support the existence of larger levels of genomic divergence than those expected for genomes pertaining to strains of similar taxonomic rank, thus providing a clear indication of the taxonomic breath of the *Acidithiobacillia* class. Consequently, we assessed the validity of the existing taxonomic assignments within the class using state-of-the-art genome-based methodologies.

### New taxonomical units of distinct rank within the *Acidithiobacillia* class

To ascertain whether strains within the assembled sets pertain to known or new taxonomic units of different taxonomic rank, OGRIs (based on amino acidic sequences) were derived from available genomic information. First, the 95 genomes recognized to belong to the *Acidithiobacillia* class in this study (Table S1b) were cross-compared in terms of the average AAI (ref. [51]) of their protein pools and 16S rRNA gene sequence identity levels (Table S3a, b).

**Fig. 1:**
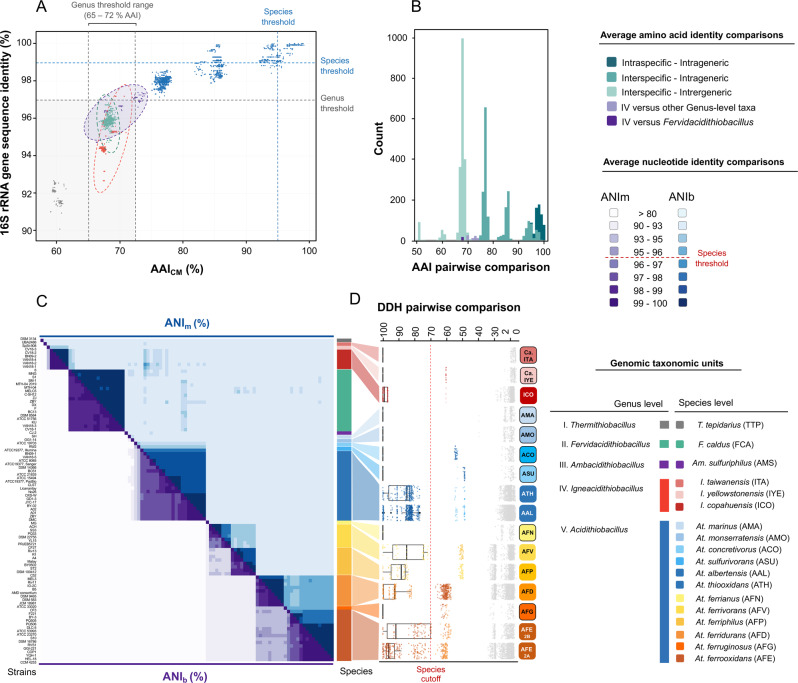
*Acidithiobacillia* class genus- and species-level taxons defined on the basis of whole-genome relatedness indexes based on amino acid and nucleotidic sequence identity pairwise comparisons. **A** Amino acid identity values calculated with CompareM (AAI^CM^; Table S3a; https://github.com/dparks1134/CompareM]) vs 16S rRNA gene sequence pairwise identities (Table S3b) showing acknowledged genus (% AAI > 70%) and species (%16 S rRNA gene > 98.7%) cutoff values [63]. **B** Histogram of AAI% values colored by the taxonomic rank of each pairwise comparison. **C** Average nucleotide identity (ANI) heatmap using ANIb and ANIm alignment algorithms threshold [47, 63]. **D** Digital DNA–DNA hybridization index (dDDH) box plot showing divergence between strains assigned to known and novel genomic species (Table S4) with respect to the species cutoff [48, 49], [red line: % dDDH > 70%]. The acronyms presented on the right have been subsequently used throughout the manuscript.

AAI values for certain pairwise comparisons surpassed the acknowledged thresholds for genus-level ranks (Fig. 1A and Table S3c) [61]. As expected, AAI values derived from the comparison of *Acidithiobacillus* species with *T. tepidarius* (inter-genus), which averaged 60.2 ± 0.1% (Fig. 1B), were lower than the genus-level threshold (acknowledged to range between 65 and 72% AAI [62]). Interestingly, AAI values between different assigned *Acidithiobacillus* spp. and strains of *A. caldus* were lower than anticipated for taxa of the same genus rank (Fig. 1A, B). The same applied to a group of six novel *Acidithiobacillus*-like isolates and two MAGs recovered from terrestrial geothermal systems (Table S3c). Finally, AAI values between different *Acidithiobacillus* species and the single strain representative of *Acidithiobacillus sulfuriphilus* [15] lie just around the acknowledged genus cutoff value, casting doubts on its actual assignment to *Acidithiobacillus* and suggesting it also constitutes a novel genus of the class (Fig. 1A, B). In sum, our comparative AAI analysis strongly suggests that the *Acidithiobacillia* class presently comprises a minimum of five distinct genera. Proposed names for the novel taxa are listed in Table 1, together with representative strains and sources of origin. To keep consistency with existing taxonomic assignments of other class members, the emended and novel genera have been named as ‘*Fervidacidithiobacillus*’ (II, ex. *A. caldus*), ‘*Igneacidithiobacillus*’ (III), and ‘*Ambacidithiobacillus*’ (IV, ex. *Acidithiobacillus sulfuriphilus*). Details on the proposed etymology, type material, and descriptions of the novel genera, including nomenclatural emendations, are detailed in Table S9.

**Table 1:** Uncovered genera forming part of the *Acidithiobacillia* class and genomic species affiliated to each genus.

	Taxonomic assignment	Type species or type strain	G + C mol% ave. ± SD (min–max)	NCBI RefSeq/GenBank accession	Source reference
*Genus-level taxa*					
I	*Thermithiobacillus*	*T. tepidarius*	66.8 (single value)	AUIS01	[7]
II	‘*Fervidacidithiobacillus*’	‘*F. caldus*’	61.4 ± 0.3 (60.9–62.1)	CP005986-9	This work
III	‘*Igneacidithiobacillus*’	‘*I. copahuensis*’	58.7 ± 0.41 (58.5–59.6)	JAAXYO00	This work
IV	‘*Ambacidithiobacillus*’	‘*Am. sulfuriphilus*’	61.5 (single value)	RIZI01	This work
V	*Acidithiobacillus*	*A*. *thiooxidans*	52.74 ± 0.12 (52.6–59.0)	AFOH01	[7]
*Species-level taxa*					
I-1	*T. tepidarius*	DSM 3134^T^	66.8	AUIS010	[11]
II-1	‘*F. caldus*’ *(ex. A. caldus)*	ATCC 51756^T^	61.4	CP005986-9	This work; [12]
III-1	Ca. I. taiwanensis	UBA2486^TS^	59.6	DDOU01	This work
III-2	Ca. I. yellowstonensis	SpSt908^TS^	59.0	DTMS01	This work
III-3	‘*I. copahuensis*’	VAN18-1^T^	58.5	JAAXYO00	This work
IV-1	‘*Am. sulfuriphilus*’ (ex. *Acidithiobacillus sulfuriphilus*)	DSM 105150^T^	61.5	RIZI01	This work; [15]
V-1	‘*A. marinus*’	SH^T^	54.3	MXAV01	This work
V-2	‘*A. montserratensis*’	GG1-14^T^	54.8	JABBOU00	This work
V-3	*A. concretivorus*	ATCC 19703^T^	52.9	JABELD00	This work; [65]
V-4	‘*A. sulfurivorans*’	RW2^T^	52.7	JAAOMP00	This work
V-5	*A. thiooxidans* subspecies *thiooxidans*	ATCC 19377^T^	53.2	AFOH01	[21]
V-6	*A. thiooxidans* subspecies *albertensis* (ex. *A. albertensis*)	DSM 14366^T^	52.6	MOAD01	This work; [22]
V-7	*A. ferrianus*	DSM 107098^T^	58.2	WNJL01	[14]
V-8	*A. ferrivorans*	DSM 22755^T^	55.5	JAAOMR00	[13]
V-9	*A. ferriphilus*	DSM 100412^T^	57.4	JAAZTY00	[25]
V-10	*A.* ‘*ferruginosus*’	CF3^T^	58.7	JABBDR00	This work
V-11	*A. ferridurans*	ATCC 33020^T^	58.1	JABBHQ00	[24]
V-12	*A. ferrooxidans* subspecies 2B	F221 (DSM 1927)^T^	58.7	JABBDP00	This work
V-13	*A. ferrooxidans* subspecies *ferrooxidans*	ATCC 23270^T^	58.8	CP001219	[23]

Uncultured taxa are indicated as Candidatus (Ca.); novel taxa described in this study are indicated by single quotation marks (‘taxa’).

*T* type strain, *TS* type by sequence.

### Novel species within the *Acidithiobacillia* class genera

We next evaluated species-level assignments within the revised *Acidithiobacillia* class (Table S4), using whole-genome similarity metrics [47–50, 63]. The ANI data, shown here as a heatmap (Fig. 1C), indicated a total of 24 clusters below the 95.9% species threshold, 18 of which also emerged from the dDDH analysis, using the 70% threshold (Fig. 1D). Therefore, several genomic species exist within the *Acidithiobacillia* class, in addition to the ten currently acknowledged species (Table 1). Other nonalignment-based indexes additionally support the majority of the clusters that were revealed from the above analysis (Table S4) [50].

With the exception of *A. albertensis*, all previously described type strains clustered apart from each other and matched independent and well-supported genomic species (Fig. 1C, D). In accordance with previous results [2, 20, 64], the analysis of genomic similarity has shown that there is insufficient divergence between the type strains of *A. albertensis* and *A. thiooxidans* to warrant their classification as separate species. Accordingly, strain DSM 14366 is recognized here as a subspecies: *A. thiooxidans* subsp. *albertensis* DSM 14366 (Fig. 1D and Table 1). Additional strains fitting the criteria of genomic subspecies (having low genomic divergence with respect to the type strains of the lineage) were identified within *A. ferrooxidans* (sublineage 2B; Fig. 1D and Table 1).

We recognized novel genomic species of the *Acidithiobacillus* genus Kelly and Wood 2000 [7], such as one *A. ferrooxidans*-related species, represented by strain CF3, and also four *A. thiooxidans*-related species, all of which have OGRIs well below the species delimitation thresholds (Fig. 1C, D and Table S4). One of the latter is strain ATCC 19703, a sulfuric acid-generating bacterium isolated from moist corroded concrete, which was originally described as the type strain of *Thiobacillus concretivorus* [65], but was later reassigned as a strain of *A. thiooxidans* [7]. Whole-genome sequence level analysis suggests this reassignment should be emended by recognizing strain ATCC 19703 as a separate species, as suggested previously by marker-based proxies [20]. Three other *A. thiooxidans*-like strains represent likely novel genomic species: strain SH (‘*Acidithiobacillus marinus*’), so named because it was isolated from sea water [66], strain GG1-14 (‘*Acidithiobacillus montserratensis*’), which was isolated from the volcanic island of Montserrat, West Indies [67], and strain RW2 (‘*Acidithiobacillus sulfurivorans*’), which was isolated from acidic ferruginous stream in Wales (Table S1a).

Despite being poorly represented in culture collections, new candidate species are present in various public datasets, as shown in Table S10. Exceptions include the genomic species ‘*Igneacidithiobacillus copahuensis*’, which is represented by at least six cultivable isolates from the Copahue Volcano and share the same 16S rRNA gene sequence oligotype as a recently reported isolate from the Italian island of Vulcano [27]. This group of strains is highly diverged from currently recognized species of *Acidithiobacillia*, having as closest relatives the SpSt908 MAG found in an acidic hydrothermal pond [28], and the UBA2486 MAG reconstructed from an acidic hotspring [29, 68], proposed here as type material for two novel candidate species Candidatus Igneacidithiobacillus yellowstonensis SpSt908^TS^ and species Candidatus Igneacidithiobacillus taiwanensis UBA2486^TS^, where TS stands for “type by sequence”. Details on the proposed etymology, type material, and descriptions of the novel species identified in this study, including nomenclatural emendations, are detailed in Table S9. This taxonomic diversity was further corroborated by the following phylogenomic analysis.

### *Acidithiobacillia* class taxa branch deep within the Proteobacteria phylum

To obtain a comprehensive phylogenetic view of the class with respect to currently acknowledged proteobacterial lineages, we reconstructed informative phylogenetic trees based on the RPs ubiquitous to both *Acidithiobacillia* and representatives of the Proteobacteria phylum. A number of MAGs tentatively assigned to the *Acidithiobacillales* order (LJTU01, LJVB01, and LJUK01) or the *Acidithiobacillus* genus (NVVQ01) in current databases were also included. Concatenated alignments of reference RPs [69] present in all genomes considered, or in a representative subset (Table S5), were analyzed using Bayesian inference (BI), ML, and the NJ approaches.

**Fig. 2:**
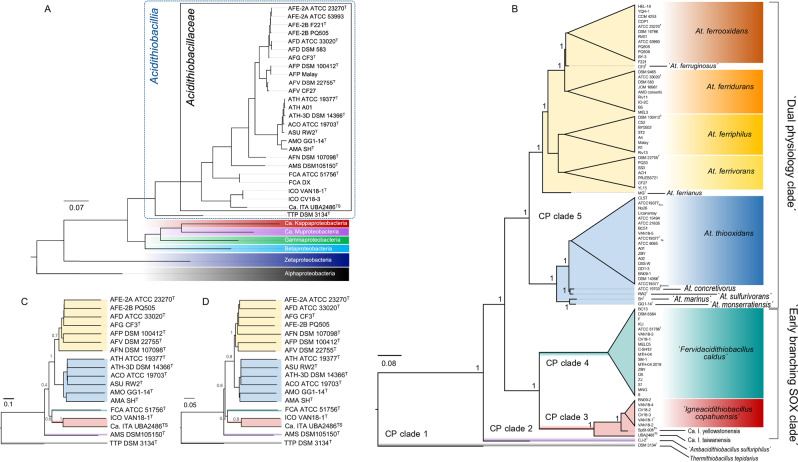
*Acidithiobacillia* class consensus phylogenetic trees built using the concatenated alignment of shared genes. **A** The MCC Bayesian tree was obtained using MrBayes with a concatenated alignment of 16 RPs (encompassing 2416 aa) that are present in all lineages and outgroups in the analysis. The equivalent tree obtained with all the 16 RPs of the *Acidithiobacillia* class is presented in Fig. S1d. *Alpha*- and *Zetaproteobacteria* form basal clades, consistent with the early branching position of these classes in the evolution of Proteobacteria [93]. **B** The ML tree was obtained with the PhyML package using 107 conserved single-copy proteins common to 88 genomes of the *Acidithiobacillia* class. Genomes of seven strains (BY-02, DMC, JYC, S10, DLC-5, GGI-221, and 21-59-9) were excluded from the phylogenomic analysis due to assembly quality issues. The alignment encompassed 24,335 aa and 10,269 parsimony informative sites. **C**, **D** Single-gene trees for RpsC (S3) and RplD (L4) constructed using NJ (10000 replicates) are shown in **C** and **D**, respectively. RPs and CPs were recovered from the genome assembly versions listed in Table S1b and detailed in Tables S5 and S6. CPs were accrued with an iterative process of filtering for genome-wise occurrence, copy number variations per genome, gene integrity, and length. Sequences aligned in this figure were reduced to simplify tree representation without altering the topology found with comprehensive alignments (Fig. S1). Branches of interest are highlighted in the tree and labeled accordingly. Posterior node support is shown as fractions. Lineages are color coded according to their taxonomic affiliation as elsewhere in the manuscript.

We found that the concatenation of a reduced set of seven fully conserved RPs (RP7) produced trees with relatively unstable posterior support using BI (Fig. S1a). Conversely, the ML approach produced informative trees with reasonable support values for the major branches (Fig. S1b). Robust and stable phylogenetic trees, encompassing all the new taxa introduced here (Table 1), were obtained using the concatenation of 16 common RP, as shown in Fig. 2A (see also Fig. S1c). Phylogenetically broad trees revealed that candidate *Acidithiobacillus* sp. NORP59 MAG (NVVQ01) [70] and candidate *Acidithiobacillales* MAGs (LJTU01, LJVB01, and LJUK01) from groundwater environments [71] did not cluster within the clade of the *Acidithiobacillia* class, but rather with representatives of the Candidate Kappa- and Muproteobacteria classes [68, 72, 73], respectively (Fig. S1a). The above-mentioned MAGs were subsequently disregarded.

With both sets of RPs, the phylogenetic trees consistently showed that the Candidate ‘*Fervidacidithiobacillus*’, ‘*Igneacidithiobacillus*’, and ‘*Ambacidithiobacillus*’ introduced here, as well as validated *Thermithiobacillus* and *Acidithiobacillus* genera, form a monophyletic clade that is sister to those of *Beta*- and *Gammaproteobacteria*. Interestingly, these clades include acidophilic iron-oxidizers, such as *Acidihalobacter* and sulfur-oxidizers, such as *Sulfuriferula* (data not shown). The deep-branching sequence of the trees supports the rank of class assigned to the acidithiobacilli by Williams and colleagues [8], and differs from the suggested position of the group as a branch of the *Gammaproteobacteria* as in v.0.1.4 of the Genome Taxonomy Data Base, GTDB [74].

### Phylogenomic analysis of the *Acidithiobacillia* class

Next, we evaluated the phylogenetic relationships between known and novel lineages of the *Acidithiobacillia* class (Table 1). To this end, we recovered over 100 CPs that were found to be common to all class species (Table S6). The phylogenetic sequence of the *Acidithiobacillia* clade varied in granular detail whether RP-based or CP-based concatenated alignments were used for tree reconstruction. RP-based trees often failed to resolve the basal position of ‘*Ambacidithiobacillus sulfuriphilus*’ (Fig. 2A), which is clearly shown by both concatenated CP trees (Fig. 2B) and single protein marker trees (e.g., Fig. 2C, D). Direct visualization of overlaid RP-based Bayesian trees revealed a broad subset of trees with different trajectories around the nodes of ‘*Ambacidithiobacillus sulfuriphilus*’ and the ‘*Igneacidithiobacillus*’ plus ‘*Fervidacidithiobacillus*’ clades, suggesting unstable segregation of the branching pattern of these taxa (Fig. S1d).

While the phylogenetic signal of concatenated RP proteins is capable to resolve only partially the most likely phylogeny of this taxon, ML and Bayesian trees reconstructed with the CP set were sufficiently resolved to show the separation of main clades and of individual new species within each clade and subclade of the acidithiobacilli (Figs. 2B and S1e). Overall, the *Acidithiobacillia* taxa listed in Table 1 form five principal clades (CP clades 1–5) coinciding with the five genera of the class acknowledged here. In the CP trees the *Acidithiobacillus*, and the ‘*Igneacidithiobacillus*’ plus ‘*Fervidacidithiobacillus*’ clades are subtended by *Acidithiobacillus sulfuriphilus*, the genome of which contains the deepest branching versions of many relevant respiratory proteins, from CtaA for heme A biosynthesis to the CyoB catalytic subunit of the most common A family oxidases of the *bo*_3_ subtype (see below). Therefore, ‘*Ambacidithiobacillus sulfuriphilus*’ (CP clade 2) can be considered to be the closest to the common ancestor of acidithiobacilli, once they diverged from the ancestor of *T. tepidarius* lineage. This possibility is further supported by the fact that ‘*Ambacidithiobacillus sulfuriphilus*’ has the highest pH optimum (3.0) and upper level of growth (7.0) of the group [15], barely crossing the pH limit set to distinguish extreme acidophiles from moderate acidophiles [16].

With the exception of the *Acidithiobacillus* CP clade 5, which includes both sulfur- and iron-oxidizing species, and is labeled here as the “dual physiology” (iron/sulfur) clade, all other clades in the CP trees are exclusively sulfur-oxidizers. The ‘*Fervidacidithiobacillus*’ (CP clade 4) and the newly identified ‘*Igneacidithiobacillus*’ (CP clade 3) contain mesothermophilic [12] or thermotolerant [14] sulfur-oxidizing taxa, respectively. Both clades appear to be early diverging (“early branching S-oxidizers”) with respect to the majority of those in the dual physiology subclade, apart from *A. ferrianus* which branches deepest within the *Acidithiobacillus* dual physiology subclade.

In partial agreement with previous reports based upon 16S rRNA gene trees, sulfur-oxidizing *Acidithiobacillus* spp. cluster in a different subclade from that containing all the iron/sulfur-oxidizing acidithiobacilli (Fig. 2B). The latter form a stable and monophyletic group, which appears to have originated before the split between the ‘*Igneacidithiobacillus*’ and the ‘*Fervidacidithiobacillus*’ clades. Such results support the genospecies uncovered in previous sections as true species, requiring formal recognition and chemotaxonomic validation.

### Pangenome analysis of the *Acidithiobacillia* class

**Fig. 3:**
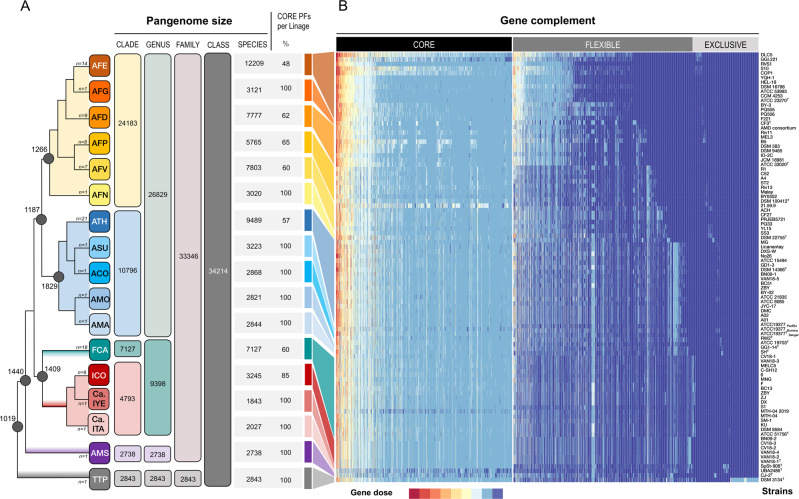
Functional analysis of the pangenome of the *Acidithiobacillia* class. **A** Dendrogram recreating the phylogenetic relations between acidithiobacilli. Terminal nodes, representing current species-level lineages are depicted as squares, and labeled with the acronym of the species names and ancestral nodes are represented by circles. Core genome feature characteristics are displayed to the right, and pangenome characteristics to the left. **B** Heatmap constructed from the genome versus function presence/absence scoring matrix. Colors reflect the dose of each gene (number of orthologs per PF identified) in each genome. Data were split between gene complement compartments (core, flexible, and exclusive) to aid visualization. Strains (per species) listed are those in Table S1.

Pangenome, core, and accessory genomes were calculated for the taxonomic ranks of the *Acidithiobacillia* class uncovered here (Fig. 3A). The size of the core genome of the different *Acidithiobacillia* taxa varied relatively little (Fig. 3A and Table S7a) and were inversely correlated with the sample size (Pearson’s correlation coefficient *r* = −0.81). For the whole class, this analysis revealed a global pangenome of 34,214 protein families (Table S7b), indicating highly diverse gene contents. The protein families encoded by an average genome (3.1 Mb) represents only 9% of the total pangenome of the class, and 46.6% (on average) of the species-level pangenomes, indicating that most acidithiobacilli have open pangenomes. Additional metrics further support this view (Table S7).

As is the case of other microbial taxa, functional analysis of core family proteins revealed a high proportion of KEGG categories associated with central or housekeeping functions (Table S7b). Sixteen percent of all protein-encoding genes varied in copy number, from 2 to 11 (Fig. 3B). Certain functional variants happened to be species-specific, as was the case of a variant ATP synthase complex in ‘*Ambacidithiobacillus sulfuriphilus*’, while others were clade exclusive, for example variant 2 of RuBisCo form I, which occurred only in the clade of iron/sulfur-oxidizing *Acidithiobacillus* spp. (not shown). In other cases, differences in gene copy numbers occurred through a group of lineages, as for cytochrome *bd* oxidase (see below). The phylogenetically resolved information derived from pangenomic analysis were used for evolutionary analysis of energy production traits (Table S7c), which ultimately determine the physiology and lifestyle of *Acidithiobacillia* taxa.

### Metabolic traits of the *Acidithiobacillia* class reflect its ancestral physiological legacy

#### Sulfur metabolism

**Fig. 4:**
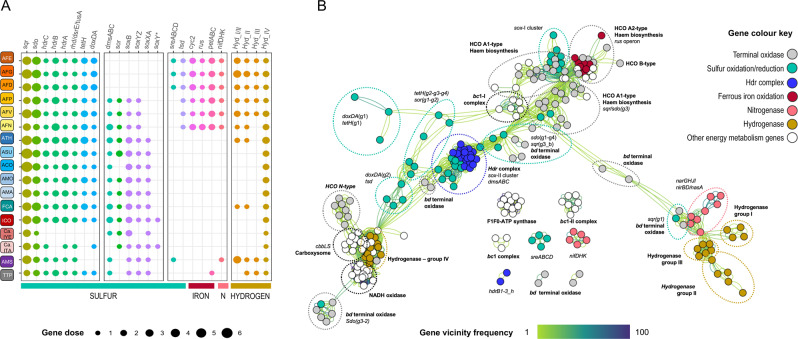
Metabolic traits of the *Acidithiobacillia* class species. **A** Phyletic patterns of relevant energy metabolism genes (and associated gene clusters). Color coding is as labeled in the figure panel. Size of the circles represents the gene dose of representative genes in each cluster. PF variants identified are detailed in Table S7c. **B** Functional association networks based on gene vicinity concurrence frequencies (edges), including variants per protein family (nodes) as detailed in Table S7c. Concurrence frequency (%) of each gene pair was scored for all genomes from gene annotation tables, at selected relevant genetic contexts. Statistical concurrence information was used to color the edges as indicated in the label and were used to derive functional modules. Additional details can be found in Supplementary Information (Extended Figure Legends).

A number of metabolic traits predicted to be involved in sulfur oxidation are conserved in most extant representatives of the *Acidithiobacillia* class. These traits include specific protein variants of a sulfide quinone oxidoreductase SQR, a sulfur dioxygenase SDO, the heterodisufide reductase complex HDR, the membrane-bound tetrathionate-forming thiosulfate:quinone oxidoreductase TQO, and the tetrathionate hydrolase TTH (Fig. 4A). Collectively, these enzymes are predicted to facilitate the utilization of hydrogen sulfide, elemental sulfur, thiosulfate, and tetrathionate as electron donors to the quinone pool of the respiratory chain of all known acidithiobacilli (Table S2).

Genes encoding the components of the *sox* thiosulfate oxidation pathway [4], present in many sulfur-oxidizing neutrophiles [75], were also found in the genomes of most *Acidithiobacillia* (Fig. 4A), except for the late-diverging iron/sulfur-oxidizers (*A. ferrooxidans*, ‘*Acidithiobacillus ferruginosus*’, and *A. ferridurans*). Partial conservation of *sox* gene cluster elements (*sox*-II: *soxYZ-hyp-soxB*) was observed in early diverging iron/sulfur-oxidizing species *A. ferrianus*, *A. ferriphilus*, and *A. ferrivorans*. However, lack of the *soxXA* cytochromes in their genomes provides evidence for the gradual degeneration of the *sox* system in iron/sulfur-oxidizers. Other sulfur-metabolizing protein family variants (of SDO, SOR, and SQR) follow the phyletic pattern of the Sox proteins in this class (Fig. 4A). On the basis of statistical concurrence information, these ancillary proteins are predicted to contribute thiosulfate to the Sox complex from other reduced sulfur sources (e.g., sulfide and/or elemental sulfur; Fig. 4B). The integrated analysis of genomic distribution and phylogenetic patterns for the proteins involved in the above-mentioned traits of sulfur metabolism sustains the concept that the last common ancestor of the class was most probably a proficient sulfur-oxidizer.

#### Iron metabolism

Redox proteins that provide the electron wiring between extracellular ferrous iron donors and the O_2_ reductase (COX) plugged in the cytoplasmic membrane, were found exclusively in *Acidithiobacillus* species of the iron/sulfur-oxidizing clade (Fig. 4A). Such proteins include the rusticyanin encoded as part of the COX operon [76] and the *pet*-I and *pet*-II operons, coding for two variants of the cytochrome *bc*_1_ complex [77, 78], which produce reduced cytochrome *c*, the substrate for COX. Membrane-bound (cytochrome Cyc2) and soluble electron carriers (*c* cytochromes) complete the respiratory chain of iron/sulfur-oxidizing acidithiobacilli [79, 80]. Their genes follow the same sequence among acidithiobacilli (Fig. 4A), suggesting that coordinated gene acquisition and/or accretion events might have occurred in the ancestor of this particular lineage. From available public information (e.g., shared traits in *Acidiferrobacteraceae* family members [81]), the most likely source of these proteins was the ancestor of iron-oxidizing *Acidiferrobacter thiooxydans* of the *Gammaproteobacteria* class.

Iron/sulfur-oxidizing *Acidithiobacillus* spp. invariably possess genes coding for nitrogen fixation (NifHDK nitrogenase and accessory proteins), sulfur reduction (SreABCD), and group 1 hydrogenases (Fig. 4A). These functions enable autotrophic growth by dissimilatory H_2_ oxidation [82] and anaerobic growth on S^0^ coupled to ferric iron reduction [83]. Among *Acidithiobacillia* taxa, these elements are present only in one other genome, the early branching ‘*Ambacidithiobacillus sulfuriphilus*’, which lacks the *rus* operon for iron oxidation (Fig. 4A). The nitrogenase and both the S^0^ reducing and the Q-reducing hydrogenases are primitive metabolic traits [84, 85], typical of anaerobic or microaerobic bacteria, frequently associated with terminal oxidases with high affinity for oxygen. This clade of the tree includes taxa that are known to be facultative anaerobes and may thus represent the relics of an ancestral state in the evolution of acidithiobacilli metabolism, with subsequent differential loss of iron metabolism and anaerobic traits.

### Terminal oxidases and the evolution of the *Acidithiobacillia* class

All members of the class posses several terminal oxidases of both high- and low-oxygen-affinity families, none of which are conserved across all the class representatives. Instead, alternative terminal oxidases showed differential patterns of occurrence, pinpointing to different events of gene acquisition/loss, diversification and, most importantly, also modular creation.

#### High-oxygen-affinity terminal oxidases

**Fig. 5:**
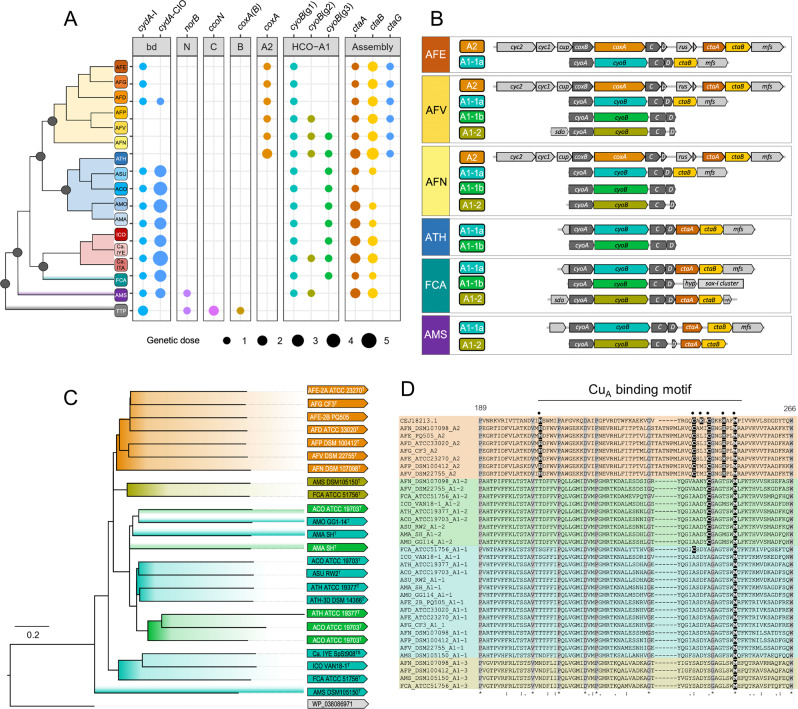
Occurrence, gene dose, and genetic context of protein families encoding electron acceptor functions in *Acidithiobacillia* class spp. **A** Phyletic pattern of terminal oxidases and relevant accessory proteins. **B** Gene neighborhood of terminal oxidases in representative species of the class. **C** Maximum likelihood phylogenetic tree of the heme A synthase CtaA genetically linked to either COX or *bo*_3_ oxidases. **D** Extract of the sequence alignment of the COX2 subunit and its ortholog CyoA, showing the copper-binding motif and its variation along acidithiobacilli taxa. COX2 protein CEJ18213 is used as reference to identify key residues of the copper-binding motif.

All genera of the class encode members of the superfamily of cytochrome *bd* ubiquinol oxidases, which allow for microaerobic respiration and enhance tolerance to several stressful growth conditions [86], ranging from two in *Thermithiobacillus* up to seven variants in certain *Acidithiobacillus* spp. (Fig. 5A). Different CIO (cyanide insensitive oxidase [87]) variants derive from multiple rounds of gene duplication and differentiation as in other bacteria [87], since their gene clusters tend to be preserved and they form part of a monophyletic clade in phylogenetic trees (Fig. S2a). The exception is represented by the single CIO of *T. tepidarius*, which clusters with homologs from sulfur-oxidizing *Betaproteobacteria*, such as *Sulfuriferula*. Moreover, the *bd*-I oxidase of *T. tepidarius* does not form part of the monophyletic clade of the *bd*-I protein subunits of all other acidithiobacilli. Notably, early diverging iron/sulfur-oxidizing acidithiobacilli (*A. ferrianus*, *A. ferrivorans*, and *A. ferriphilus*), which are typically slow-growers [13], completely lack the genes for *bd* oxidases; their function seems to have been compensated by one or more ubiquinol oxidases of the cytochrome *bo*_3_ subtype of A family HCO—heme copper oxygen reductases (Fig. 5A). *Thermithiobacillaceae* additionally have two representatives of C family HCO (often called *cbb*_3_ oxidases [88]), which are unique to the whole class (Fig. 5A).

#### Other terminal oxidases

Most likely, A family HCO, which is considered to have relatively low affinity for oxygen [89], appeared in the *Acidithiobacillia* class after branching from the *Thermithiobacillaceae* (Fig. 5A). Most common in *Acidithiobacillia* are variants of the A1 subtype of cytochrome *bo*_3_ ubiquinol oxidases, which fall into two groups that we named A1-1a/A1-1b and A1-2 on the basis of their segregation into different clades within phylogenetic trees reconstructed with their CyoB COX1 subunit (Fig. S2b) or CyoA (COX2 ortholog) subunit (data not shown). The A1-1a subvariant is present in all species of the *Acidithiobacillaceae* family, while the A1-1b subvariant appears after the branching point that gave rise to the *Ambiacidithiobacillus* spp. (Fig. 5A), and is lost in late-branching iron/sulfur-oxidizers, as does its genetically linked *sox*-I gene cluster (Figs. 4A and 5B). The A1-2 variant showed an uneven pattern of distribution in the class, with genomic evidence of horizontal gene transfer (HGT) dispersion in certain extant lineages [31].

Both the A1-1a and A1-2 variants are encoded by gene clusters, including the genes for the concerted biosynthesis of heme A, *ctaB* (heme O synthase), and *ctaA* (heme A synthase; Fig. 5B). The presence of the gene for CtaA is unusual, since it is not observed in the gene cluster of cytochrome *bo*_3_ ubiquinol oxidases of other bacteria, which often terminate with a CtaB homolog called CyoE [5]. Considering that A2-type *aa*_3_ cytochrome *c* oxidases (COX) are not present in the genomes of extant sulfur-oxidizing acidithiobacilli, we surmise that such bacteria possess an “A1-type” *aa*_3_ ubiquinol oxidase, as supported by biochemical and spectral evidence reported previously [90]. This in turn suggests that the usual definition of cytochrome *bo*_3_ ubiquinol oxidases is biochemically incorrect for the terminal oxidases of this subtype present in *Acidithiobacillia*, also because it is possible that such enzymes may have a dual function of oxidizing either ubiquinol or soluble *c* cytochromes [91].

Phylogenetic analysis has shown that the predicted CtaA products of genes found in the immediate vicinity of the A2-type and the A1-2-type oxidases are more closely related to each other than to the A1-1a/A1-1b oxidases (Fig. 5C). Among the latter, CtaA (linked to the A1-1a subtype) of ‘*Ambacidithiobacillus sulfuriphilus*’ and ‘*Fervidacidithiobacillus caldus*’ are the deepest branching among those encoded in the genomes of acidithiobacilli. This is not the case of the catalytic subunits CyoAB of the congnate operons, which are late diverging with respect to their COX orthologs (data not shown; see [92] for preliminary results). This evidence strongly supports modular construction of chimeric oxidases in the *Acidithiobacillia* class.

The CyoB and CyoA subunits seem to have evolved from an ancestral form of the A2-type oxidases into representatives of the cytochrome *bo*_3_ subtype of the A family HCO. The most conspicuous change in this evolution has been the loss of the binuclear Cu_A_ center, which is bound to the COX2 subunit via six conserved amino acids [88]. These residues include a pair of cysteines that are normally missing in the CyoA subunit of the cytochrome *bo*_3_ subtype. Uniquely among bacterial oxidases, CyoA proteins of ‘*F. caldus*’ and *A. thiooxidans* (A1-1a variant) still retain one or the other of such cysteines (Fig. 5D), thereby representing a missing link in the transition between cytochrome *c* oxidases into *bo*_3_ ubiquinol oxidases of the A family (cf. ref. [5]). A similar presence of vestigial cysteines has been previously documented for the NuoD subunit in complex I, which in some bacteria retain vestigial Cys ligands of the Ni–Fe cofactor of their hydrogenase ancestors [87]. The combination of this unique feature with the previously mentioned phylogenetic properties suggests that extant sulfur-oxidizing acidithiobacilli retain genomic and molecular remnants of the evolutionary transition of COX into Cu_A_-lacking *aa*_3_ (ubiquinol) terminal oxidases. As such remnants have not been found in other bacterial lineages, it follows that *Acidithiobacillia* might be the originators of *bo*_3_ ubiquinol oxidases, a hypothesis we will pursue in the immediate future.

## Discussion

Using diverse complementary approaches and an extensive set of genomes, we present here the most comprehensive study to date of the taxonomic and phylogenetic structure of the *Acidithiobacillia* class. Novel results supported the assignment of *Acidithiobacillus sulfuriphilus* strain CJ-2, *A. caldus* strains, and *Acidithiobacillus*-like spp. from the Copahue Volcano and similar environments to three different prokaryotic genera (‘*Ambacidithiobacillus*’, ‘*Fervidacidithiobacillus*’, and ‘*Igneacidithiobacillus*’), which are well separated from known *Acidithiobacillus* taxa, as well as *Thermithiobacillus*. Our analysis also revealed the existence of several novel species of acidithiobacilli, which significantly expands the genomic diversity of this class. Importantly, the phylogenomic analysis described here has provided a novel and robust framework for understanding the eco-physiology and evolution of extant species of the class. For example, it has indicated the deep-branching position of *A. ferrianus* among iron/sulfur-oxidizing acidithiobacilli (cf. Fig. 2), suggesting that the currently available genomes may only partially cover the phylogenetic space of these extremophiles. Indeed, the functional profile of iron/sulfur-oxidizing acidithiobacilli partially overlaps that of their deepest branching relative, ‘*Ambacidithiobacillus sulfuriphilus*’, especially in regard to traits of energy conservation, such as the combined presence of type 1 (ubiquinone-reducing) membrane hydrogenases and nitrogenase (cf. Fig. 4). Such traits are present also in *Gamma*- and *Betaproteobacteria* with sulfur-oxidizing physiology [93], which form part of the sister clades of *Acidithiobacillia* (Fig. 2). Such clades clearly evolved after separation from the *Alphaproteobacteria* lineage, which is recognized as the deepest branching class of Proteobacteria [94]. By inference, the class *Acidithiobacillia* is among the basal lineages of Proteobacteria.

**Fig. 6:**
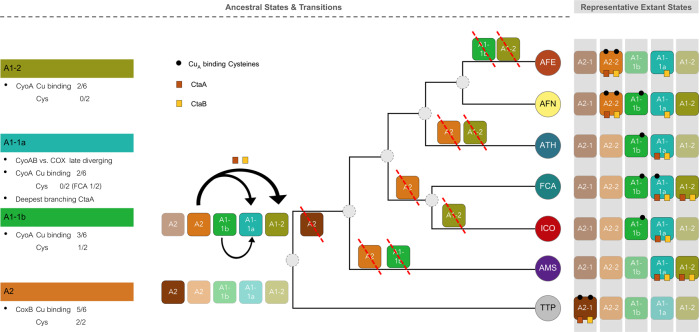
Emerging evolutionary scenario for heme copper oxidases (HCO) in the *Acidithiobacillia* class. Two variant A2-type (COX) and three variant A1-type (CYO) oxidases were identified in the genomes of sequenced acidithiobacilli. Left, characteristics of extant A1-2, A1-1a/1b, and A2-type HCO CoxB and CyoA subunits with respect to the conservation of the Cu_A_ biding motif (number of conserved residues of the six-residues Cu_A_-binding site and the number of conserved cysteines in the CXXXC moiety). The A1-1a variant gene clusters are unusual in that they group together (in the same gene cluster) the deepest branching CtaA heme A synthase-encoding genes, with genes encoding late-diverging CyoAB structural subunits. Right, evolutionary transitions inferred for the HCO terminal oxidases of the *Acidithiobacillia* class, based on the phyletic patterns of both COX/CYO and CtaAB-encoding genes in extant lineages of the *Acidithiobacillia* class. Only representative species-specific patterns are depicted for simplicity. Colored circles identify the lineages by the acronyms, and colored boxes the HCO variants present in each lineage. Fully colored boxes indicate presence and opaque boxes, absence. Boxes crossed with red lines along the dendrogram, represent probable instances of gene loss in ancestral branches of the *Acidithiobacillia* class tree. The dendrogram is based on CP tree depicted in Fig. 2. Conservation of the cysteins of the CXXXC Cu_A_-binding motif and gene context-based association with heme A biosynthesis genes is symbolized in the right panel accordingly.

On the basis of the growth optima and tolerance ranges of the deepest branching taxa, it may be inferred that the last common ancestor of the *Acidithiobacillia* class was a thermophile. Present-day strains of *T. tepidarius* and ‘*F. caldus*’ can endure temperatures well >50 °C [11, 12], while accumulating evidence suggests that ‘*Igneacidithiobacillus*’ spp. are also thermotolerant (up to 40 °C [27, 95]). Quite probably, this ancestor was a neutrophile as is present-day *T. tepidarius* (optimal pH 7 [11]), which gradually developed acid tolerance much like ‘*Ambacidithiobacillus sulfuriphilus*’ (with a reported pH growth range between 1.8 and 7.0 [15]). Sulfur oxidation can be considered the most common physiology across the whole class of *Acidithiobacillia*, the ancestor of which were probably microaerophilic as extant members of the *Thermiacidithiobacillus* genus, which have terminal oxidases with high affinity for oxygen, such as the *cbb*_3_ oxidase (Fig. 5). These bacteria, however, are unlikely to represent ancestral models for extant acidithiobacilli because their terminal oxidases, as well other traits of energy metabolism, are close to those of sulfur-oxidizing *Betaproteobacteria* and unrelated to those of other members of the class (Fig. 5 and data not shown). Conversely, the metabolic trait of iron oxidation is restricted to a specific clade of *Acidithiobacillia*, supporting the possibility that iron chemolithotrophy might have arrived late in the evolutionary history of the class, acquired by HGT from other primitive iron-oxidizing microorganisms. However, it remains intriguing that the same taxa possessing the aerobic traits of iron oxidation are the only facultative anaerobes in the class [26] and have the elements of nitrogen fixation, which is an ancient trait characteristic of anaerobic bacteria. The presence of several of these ancient traits in deep-branching ‘*Ambacidithiobacillus sulfuriphilus*’ (e.g., the nitrogenase) supports the possibility that some of them were present already in the common ancestor of the acidithiobacilli, after branching of the lineage of extant *Thermothiobacillus*. Subsequent differential loss might then explain their patchy distribution in current taxa (Fig. 6), as has occurred in other taxa exposed to strong selective conditions during their evolutionary course (e.g., sulfur-oxidizing symbionts of *Gammaproteobacteria* [93]). While initially iron reduction may have provided the ecological advantage for retaining nitrogen fixation under anaerobic conditions, active oxygen consumption achieved through iron oxidation may still provide local situations of virtual anaerobiosis favoring the function of oxygen-sensitive nitrogenases. Such bacteria were almost certainly aerobes (or facultative anaerobes) and capable of bioleaching trace metals associated with the abundant pyrite minerals that were present on the exposed continental crust. Therefore, such bacteria were the likely progenitors of extant iron/sulfur-oxidizing acidithiobacilli.

Detailed analysis of energy conversion traits that we present here suggests that a long missing transitional state in the evolution of extant A1-type *bo*_3_ quinol oxidases form A2-type *aa*_3_ HCO oxidases has been captured in the *Acidithiobacillia* class. Following this finding, we propose a probable evolutionary scenario for these terminal oxidases. In response to bursts of cyanobacteria-produced oxygen in emerged lands of primordial earth [96], ancestors of extant acidithiobacilli (presumably microaerophilic sulfur-oxidizers), evolved or acquired a primordial A2-type cytochrome *c*-oxidase (“A2-COX-*aa*_3_”)—including its assembly factors CtaA and CtaB. Shortly after, the capacity to oxidize iron, in addition to sulfur, provided an initial ecological advantage for ancestral acidithiobacilli. Later on in their evolution, depletion of pyrite and other sulfidic minerals, and reduction in ambient oxygen levels [96] promoted the transition of cytochrome *c*-oxidase into a Cu_A_-lacking ubiquinol oxidase, still retaining heme A (“A1-COX-*aa*_3_”). This new ubiquinol oxidase, became the dominant terminal oxidase after the loss of the cytochrome *bc*_1_ complex, no longer necessary for driving energy production via reverse electron transfer [78]—once sulfur compounds became the major electron donors to the respiratory chain. Subsequently, loss of heme A biosynthesis and complete degeneration of the Cu_A_-binding motif, brought about the transition of the A1-COX-*aa*_3_ into the A1 *bo*_3_ quinol oxidases found today in most Proteobacteria.

In conclusion, this study has significantly expanded the genomic countours of the *Acidithiobacillia* and highlighted important lacunae in our comprehension of the phylogenetic space occupied by the class. The data and conclusions call for further exploration of unconventional environments, including those that may be considered relics of the Earth’s past, and for additional genomic studies of the *Acidithiobacillia*. The phylogeny obtained thus far provides grounds to delineate the likely evolutionary scenarios of these extremophiles and further completes the emerging pamorama on the evolution of the *phylum* Proteobacteria.

## Supplementary information

Supplementary Information

Table S1

Table S2

Table S3

Table S4

Table S5

Table S6

Table S7

Table S8

Table S9

Table S10

## Data Availability

Draft genome sequences were deposited at the GeneBank database under the accession IDs and the whole-genome shotgun project accession numbers summarized in Table S1b. The scripts and pseudocode to parse tables, order data, recover, and score specific results alluded in this work are available at https://figshare.com/s/460559b9ad6b816ec649.
