# Supplementary material for: Genomic evolution of the class Acidithiobacillia: deep-branching Proteobacteria living in extreme acidic conditions
Source: ISME J. 2021 May 18;15(11):3221–38. doi: 10.1038/s41396-021-00995-x (PMC8528912; doi:10.1038/s41396-021-00995-x)
Supplement: Supplementary file 1 — Supplementary Information [file 41396_2021_995_MOESM1_ESM.docx]

**Genomic evolution of the class *Acidithiobacillia*: deep branching Proteobacteria living in extreme acidic conditions**

Ana Moya-Beltrán^1,2,3^, Simón Beard^1,3,4^, Camila Rojas-Villalobos^1,3^, Francisco Issotta^1,2^, Yasna Gallardo^1^, Ricardo Ulloa^5^, Alejandra Giaveno^5^, Mauro Degli Esposti^6^, D. Barrie Johnson^7^*, Raquel Quatrini^1,3,4^*

^1^ Fundación Ciencia & Vida, Avenida Zañartu 1482, Ñuñoa, 7780272, Santiago, Chile.

^2^ Facultad de Ciencias de la Vida, Universidad Andres Bello, Santiago, Chile

^3^ ANID – Millennium Science Initiative Program – Millennium Nucleus in the Biology of the Intestinal Microbiota, Santiago, Chile

^4^ Facultad de Medicina y Ciencia, Universidad San Sebastián, Providencia, 7510157, Santiago, Chile

^5^ PROBIEN (CCT Patagonia Confluencia-CONICET, UNCo), Departamento de Química, Facultad de Ingeniería, Universidad Nacional del Comahue, Neuquén, Argentina

^6^ Center for Genomic Sciences, Universidad Nacional Autónoma de México (UNAM), Cuernavaca, México

^7^ School of Biological Sciences, Bangor University, Bangor LL57 2UW, UK, and Faculty of Health and Life Sciences, Coventry University, Coventry, CV1 5FB, UK

* Correspondence

Raquel Quatrini, Phone: +56 22 367 2044, Mail: rquatrini@cienciavida.org

D. Barrie Johnson, Phone: + 44 1248 382358, Mail: : d.b.johnson@bangor.ac.uk

**Additional information**

Additional information supporting and expanding the results presented in this study are provided as Supplementary Tables (10 display items) and Supplementary Figures (2 display items). Detailed legends are presented below and included in each display item.

**Table S1.** Strains and genomes used in this study and associated metadata. (**A**) *Acidithiobacillia* class strains used in the study and metadata of origin. Data were recovered from listed publications, genome submission reports and/or collected in this study. When not reported specifically in the literature, latitude, longitude and altitude were calculated for the city, province or prefecture available. Sample type was normalized as follows: water (water, drainage, acid mine drainage), slurry (mud, sediment), soil (soil, ash). (**B**) *Acidithiobacillia* class genomes used in the study, accession identifiers and metadata of sequencing processing. Data were recovered from genome submission reports, listed publications and/or collected in this study. Sequencing and assembly methods and statistics are listed accordingly. The public genomes of the following *Acidithiobacillia* class species type strains were used as references: *At. albertensis* DSM 14366^T^, *At. caldus* ATCC 51756^T^*,* *At. ferrianus* DSM 107098^T^, *At. ferridurans* JCM 18981^T^, *At. ferrooxidans* ATCC 23270^T^, *At.. sulfiriphilus* DSM 105150^T^, *At. thiooxidans* ATCC 19377^T^ and *T. tepidarius* DSM 3431^T^. Missing genomes for the type strains of the *At. ferriphilus* DSM100412^T^ and *At. ferrivorans* DSM 22755^T^ have been generated in the present study. All available *Acidithiobacillus* strains and the single sister-clade sequenced representative of *Thermithiobacillus, T. tepidarius*, were classified in phylogenetic groups utilizing their 16S rRNA gene sequences, and oligotyped as described by Nuñez and colleagues [20]. ND: no data.

**Table S2.** Experimentally validated electron donors and acceptors, and growth conditions for members of the *Acidithiobacillia* class.

**Table S3.** Relatedness of *Acidithiobacillia* class members inferred from genomic data. (**A**) Relatedness inferred from amino acidic data using alignment-based methods. Average amino acid identity (AAI) as calculated using the CompareM calculation method ([https://github.com/dparks1134/CompareM], AAI^CM^) and the aai.rb implementation from the Kostas Lab [51], downloaded from github (<https://github.com/lmrodriguezr/enveomics>; commit signature: fae592f) and run using default parameters. (**B**) Relatedness inferred from 16S rRNA gene sequence identities. 16S rRNA genes were recovered from the GenBank annotation of each of the genomes under comparison and their pairwise identities calculated using the BLASTn alignment algorithm, bidirectionally and averaged. (**C**) Average amino acid identity basic statistics for intra-specific and inter-generic comparisons of *Acidithiobacillia* class taxa. (**i**) Intragroup comparisons between acknowledged species. (**ii**) Inter-group comparisons of acknowledged *Acidithiobacillia* class genera and novel candidate genera. Average amino acid identity between any two individuals of well-known species groupings such as *At. ferridurans, At. ferriphilus* or *At. caldus* (intra-group or self-comparisons) ranged between 96.7 and 100%, confirming the validity of strain to species assignments (species-level AAI% cut-off threshold: >95 [62]). The intraspecies AAI levels between strains ascribed to *At. ferrooxidans, At. ferrivorans* or *At. thiooxidans* crossed the species cut-off threshold, indicating the presence within these groups of significantly differentiated strains that most likely represent additional cryptic species, in addition to a number of mis-assigned taxa (see Table S1). AAI values for pairwise comparison between known *Acidithiobacillus* species representatives (intra-genus and inter-specific) were generally lower than 95% (averaging 79.6 ± 11.1%).

**Table S4**. Relatedness of *Acidithiobacillia* class members inferred from nucleotide data using alignment-based methods (dDDH, ANIb, ANIm) and alignment-independent methods (TETRA, TZMD). Type strains are marked with a TY superscript. Thresholds used for species delimitation are the following: digital DNA:DNA hybridization dDDH>70% (same genomic species [48, 49]); Average Nucleotide Identity ANI > 96% (same genomic species [47, 63]); Tetranucleotide frequency correlation coefficients TETRA > 0.999% (same genomic species^41^); Tetranucleotide-derived Z-value Manhattan Distance TZMD > 0.21% (same genomic species [50]). A summary of the genome relatedness indexes per genomic species pair for informative species pairs is also provided.

**Table S5.** Ribosomal proteins present in genomes and MAGs of the *Acidithiobacillia* class. (**A**) Protein IDs of the RP orthologs in representatives of the acidithiobacilli and proteobacterial lineages used in phylogenetic analysis. A number of MAGs found in databases with tentative assignments to the *Acidithiobacillales* order (LJTU01, LJVB01, LJUK01) or the *Acidithiobacillus* genus (NVVQ01) were also included. RP families used in the concatenate for construction of phylogenetic trees RP7_tree and RP16_tree are indicated [69]. **(B**) Presence/absence matrix of the different RP orthologs in the 95 genomes of acidithiobacilli.

**Table S6.** Conserved protein sequences recovered from the pool of orthologs common to all species of the *Acidithiobacillia* class used in the concatenate for construction of the CP-tree (Fig. 2b).

**Table S7.** Pangenome analysis of the *Acidithiobacillia* class. (**A**) Summary of pangenome metrics for *Acidithiobacillia* class lineages based on Tettelin´s model [54] for lineages with more than five genomes. (**B**) Proteins per protein family (PF) scored in the *Acidithiobacillia* class genomes classified by function (KEGG) and gene complement compartment (1_core; 2_flexible; 3_exlcusive), including data on occurrence (# of genomes having an ortholog for each KEGG) and total gene counts (# of orthologs for each KEGG in the whole dataset). (**C**) Metabolic features/traits scored in the *Acidithiobacillia* class, including protein family identifiers (KEGG), inferred roles and reactions (EC number), and the PF variant IDs of each gene product. Membership of the genes of interest to specific gene clusters is also indicated.

**Table S8.** Genomic variability of sequenced representative strains of sampled *Acidithiobacillia* class lineages. G+C mol%, # of CDSs and size variations between strains of each species/lineage (intra-clade) and between strains of a lineage and the reference type species of the genus (*At. thiooxidans*) are shown. Detailed information available in Table S1.

**Table S9.** Supporting information on the novel taxa described in this study. Etymology for known, novel and emended genera and species of the *Acidithiobacillia* class. Type material identifiers (type strain and genome accession numbers). Relevant phenotypic aspects of the described taxa including, cellular morphology, motility and lifestyle.

**Table S10.** Sequences assigned to novel *Acidithiobacillia* class spp. identified in this study with representatives in public nucleotide sequence databases: (**A**) 16S rRNA gene sequences; (**B**) Selected HKGs; (**C**) HKGs concatenate or (**D**) metagenomic datasets. In the case of metagenomic samples, reads were scored as in Nuñez and colleagues [20] and the derived information analyzed in the context of strain-specific data. Metadata extracted from each sequence deposit are also shown, including information on: the microorganism (species, strain, culturing status), the sample (origin, source, type, ore, location), and the inferred phylogeny and typing of each entry (clade, subclade, oligotype). In the case of 16S rRNA gene sequences oligotype is defined as in Nuñez and colleagues [20]. Blue highlights indicate the type strains of already validated species.

**Figure S1.** Supporting phylogenetic trees for the RP- and the CP-concatenated sets of proteins of the *Acidithiobacillia* class. (A) Bayesian phylogenetic tree and (B) Maximum Likelihood (ML) inference tree using 7 Ribosomal protein families (RP7_tree: S8, S17, L5, L14, L15, L16, L18) present in all lineages and proteobacterial outgroups in the analysis (Table S5). The alignment encompassed 924 aa and contained 224 conserved sites. (C-D) Bayesian tree obtained with a larger set of concatenated RP proteins of the *Acidithiobacillia* class (RP16_tree, extended supporting tree for that in Fig. 2a). (C) Overlaid single gene (RP1 to 16) Bayesian trees are shown in mirror image as displayed by the DensiTree program. RPs were recovered from the genome assembly versions listed in Table S1b). (D) Full visualization of a large set of concatenated RP (RP1 to 16) proteins using 85 strains of *Acidithiobacillia* and 8 representative Proteobacteria. (E) Bayesian tree of Conserved proteins ML (CP_tree) using 107 conserved single copy PF’s present in all *Acidithiobacillia* class lineages, represented in the analysis by 88 genomes. The alignment encompassed 24335 aa and contained 24239 positions without gaps and 10269 parsimony informative sites. These CPs were accrued with an iterative process of filtering for genome-wise occurrence, copy number variations per genome, gene integrity and length. The final set included 107 complete protein sequences that are shared by 88 representative genomes assigned to the *Acidithiobacillia* class (Table S6).


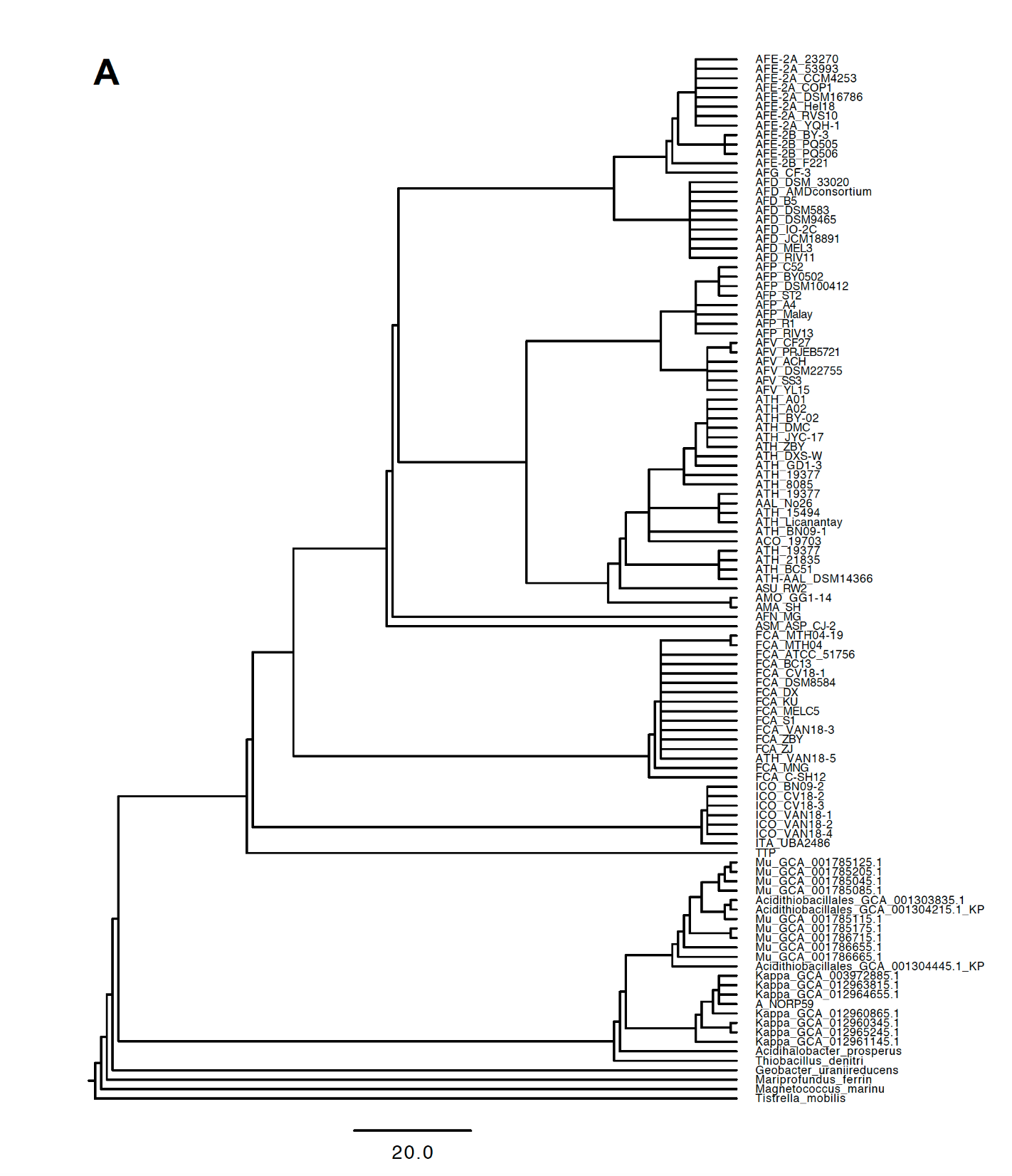


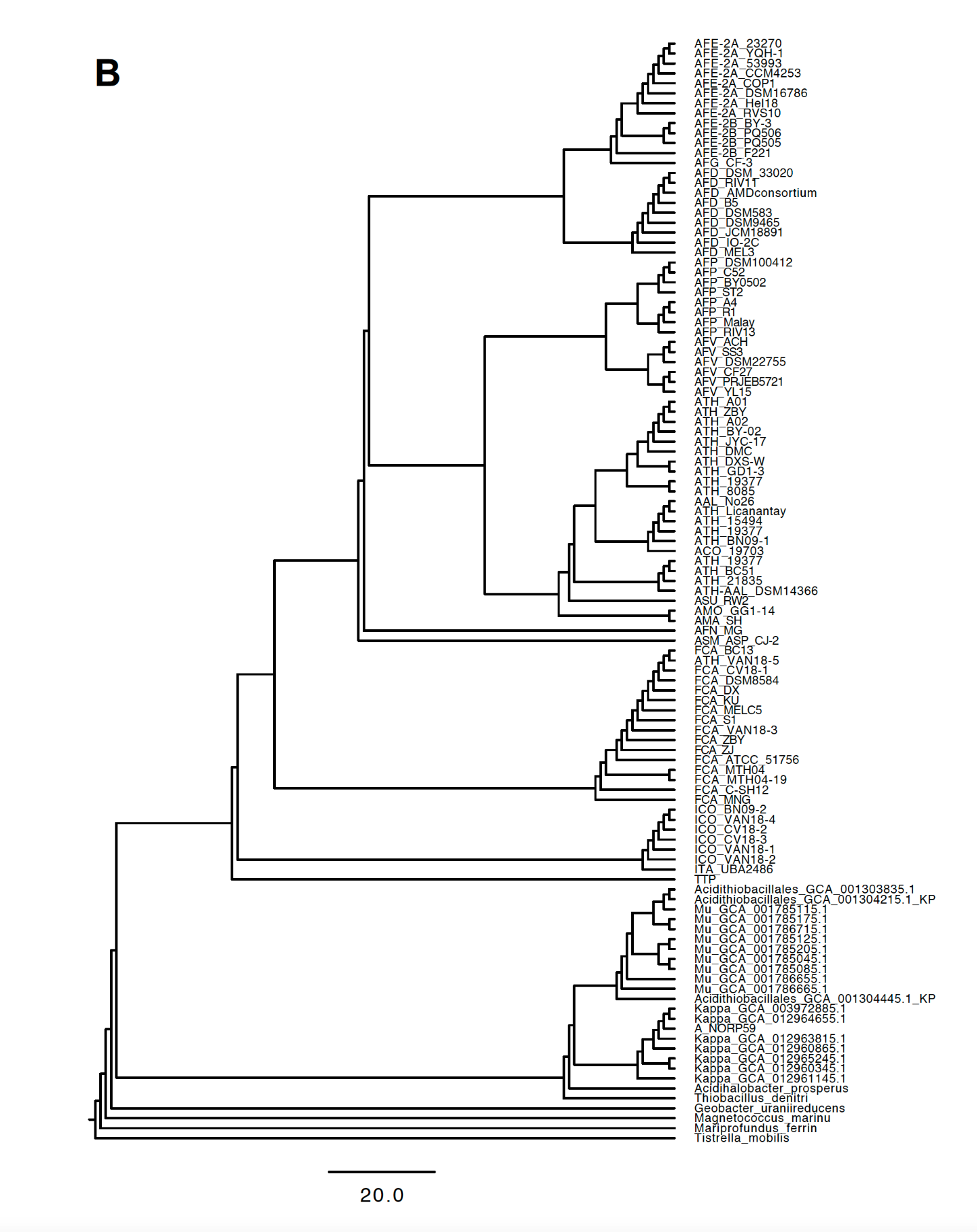


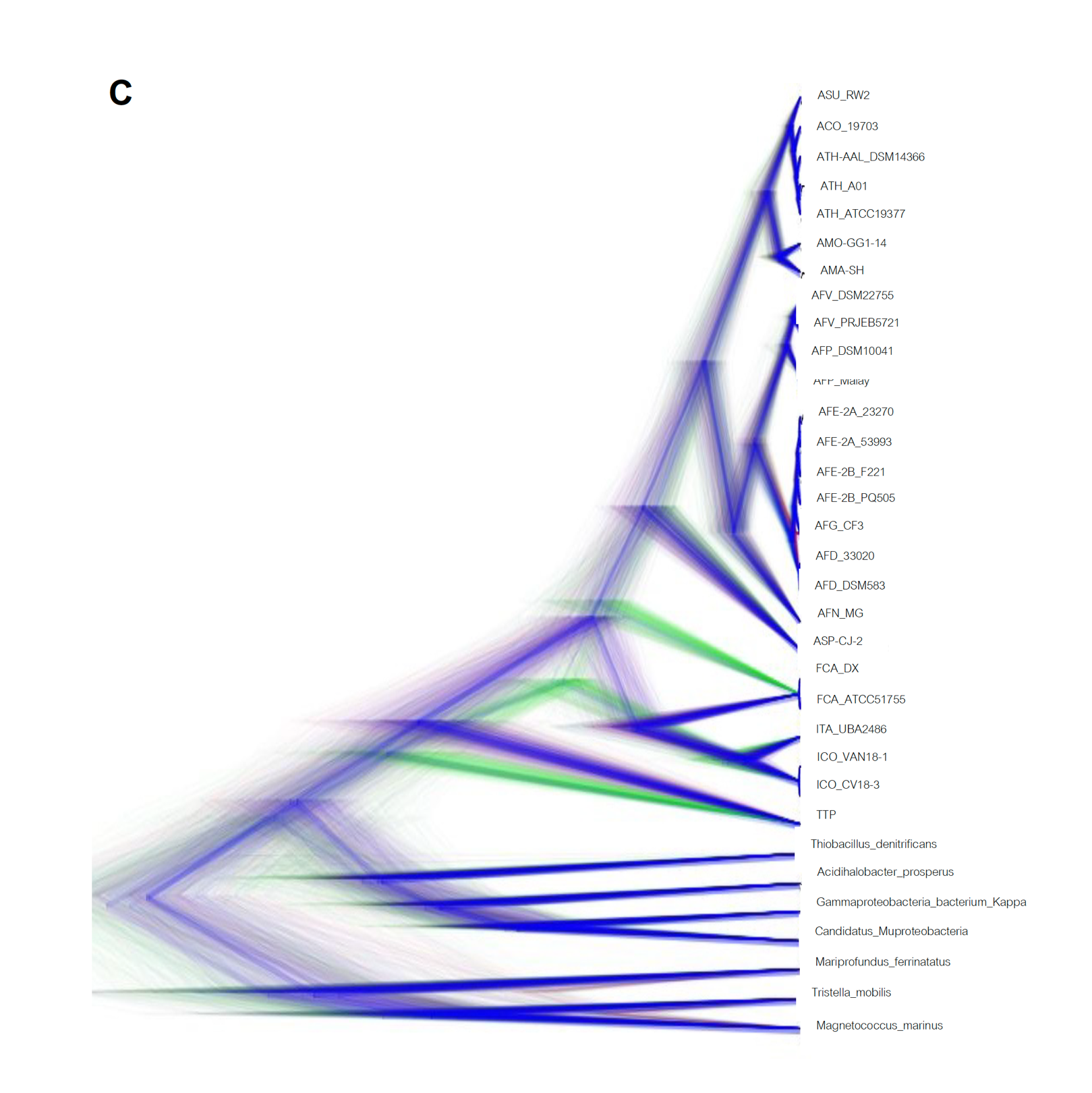


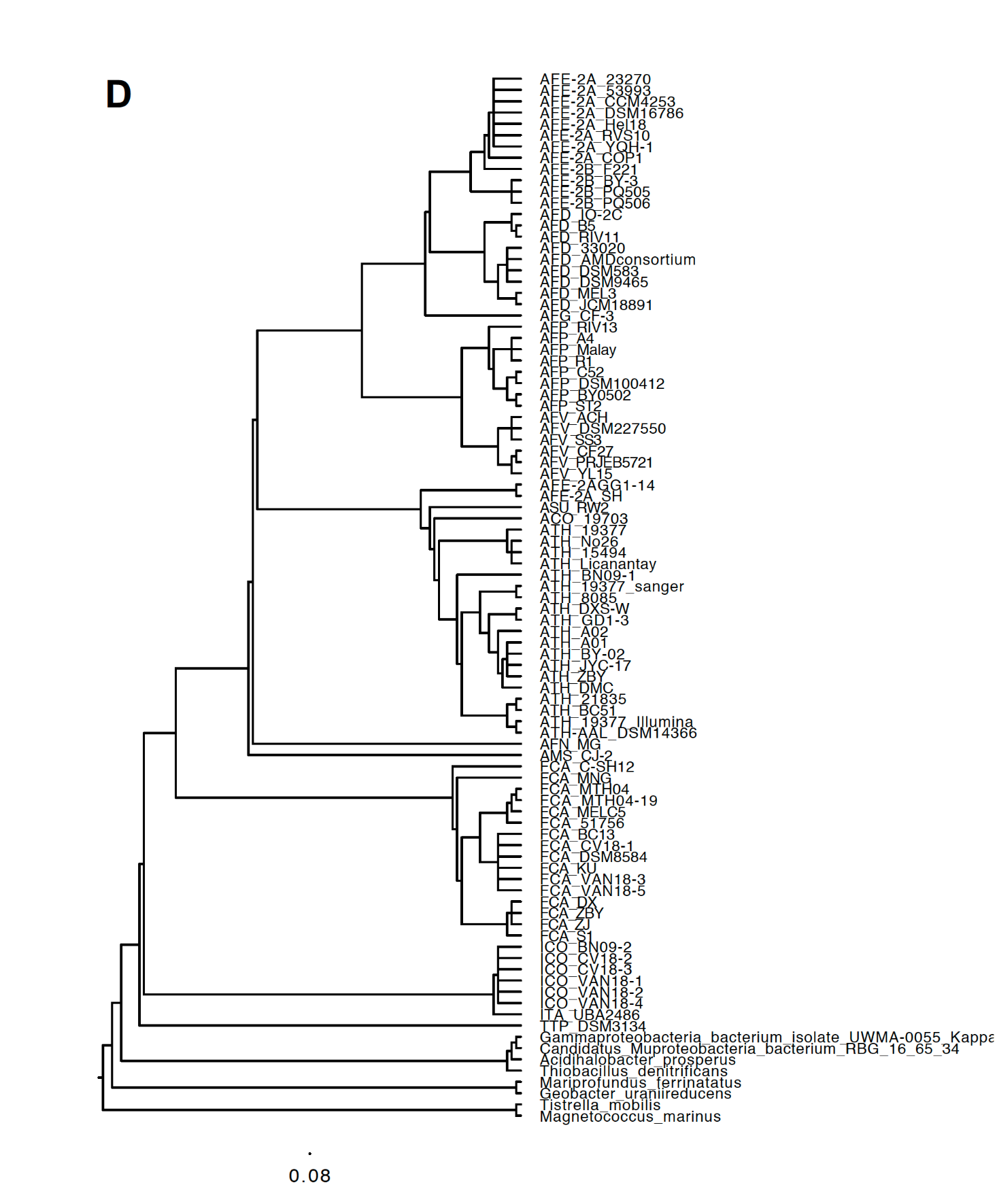


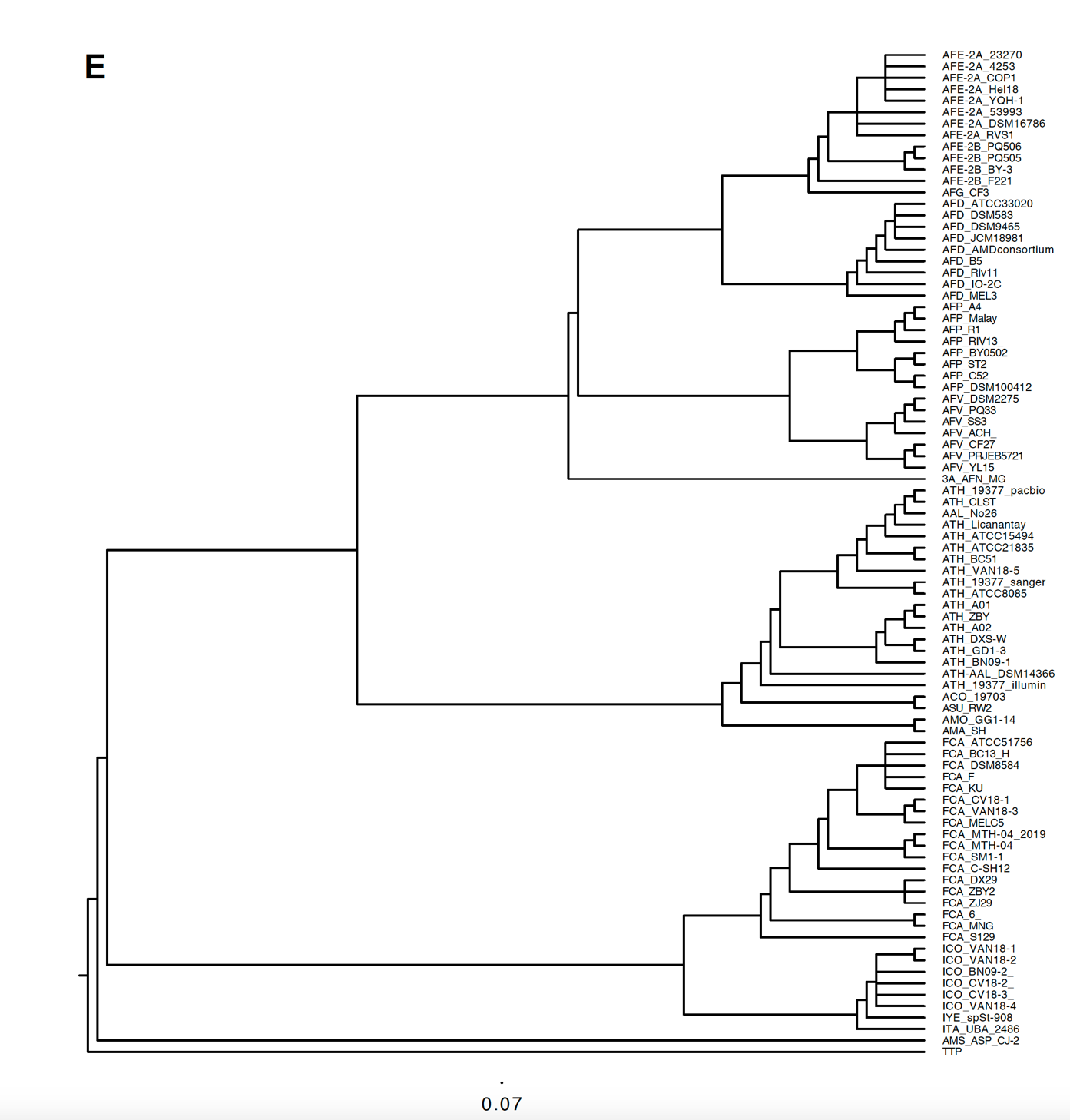


**Figure S2.** Supporting phylogenetic trees for terminal oxidases subunits of the *Acidithiobacillia* class. (**A**) Bayesian phylogenetic tree of the *bd* oxidase CydA subunit present in all lineages. The figure shows two distinct clades grouping the *bd*-I oxidases or  the *bd*-CIO oxidases (7 variants) found in the *Acidithiobacillia* class representatives. With the exception of *Thermithiobacillus tepidarius,* which branches apart from the other acidithiobacilli CIO oxidases, and together with *Thiomonas intermedia* (WP_013122030), *Metallibacterium scheffleri* (WP_081129711) and *Sulfuriferula multivorans* (WP_124703210). *Acidiferrobacteraceae* bacterium (MBP80832), *Acidiphilium multivorum* (GAN73746) and *Salinisphaera halophila* (ROO31855) were used as outgroups. The phylogenetic tree was constructed using a WAG model on a 360 aa alignment, with 333 parsimony sites. (**B**) Bayesian phylogenetic tree of the A1-type ubiquinol oxidase subunit I variants (CyoB) recovered from *Acidithiobacillia* class representatives. Three clades are evident A1-1a, A1-1b and A1-2. Other A1-type reference sequences included in the tree belong to *Acidiphilium* sp. (WP_011941987) and *Salinisphaera hydrothermalis* (WP_084189164). The tree was built from a 710 aa alignment, having 582 parsimony sites.

**
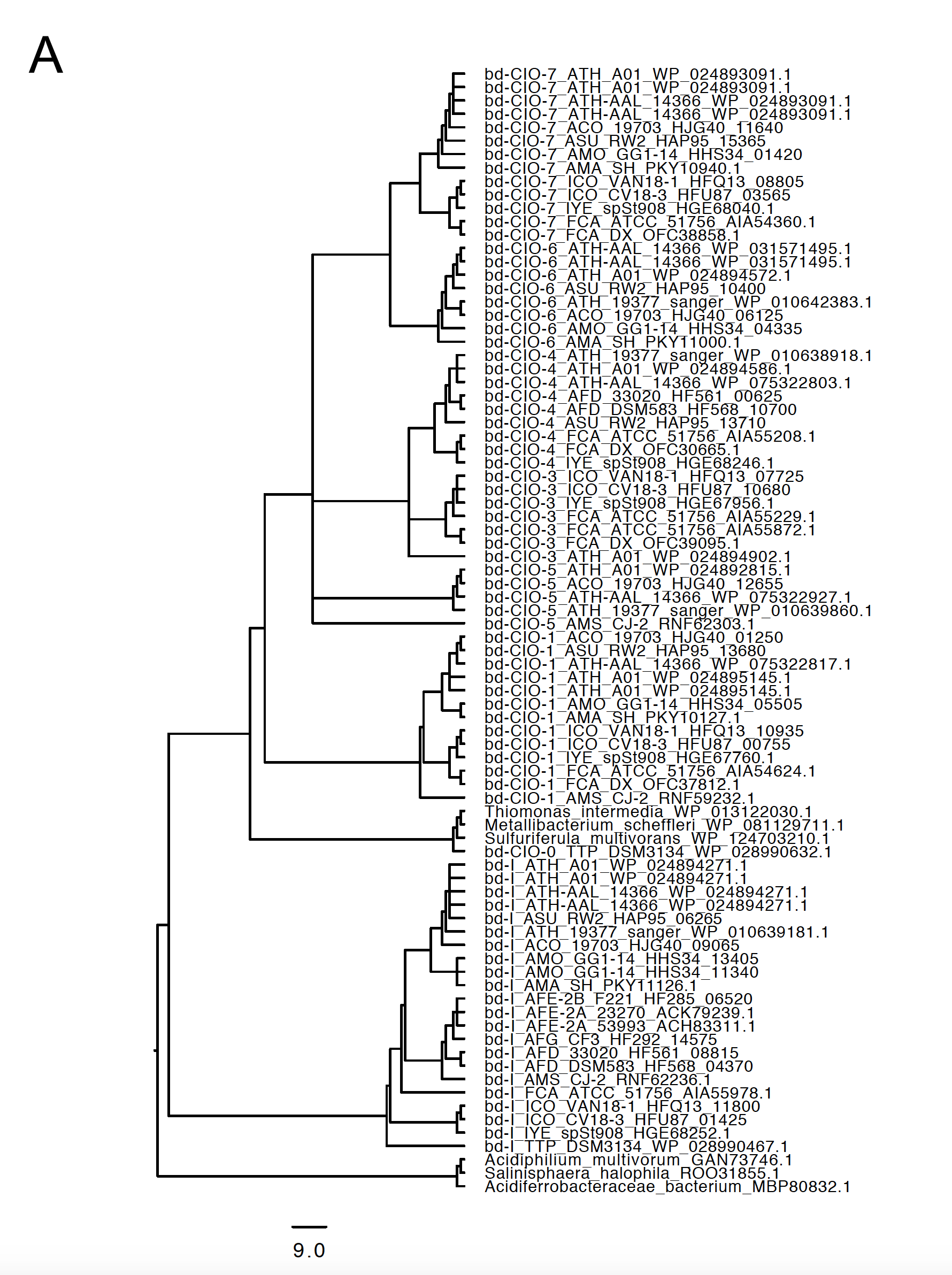
**

**
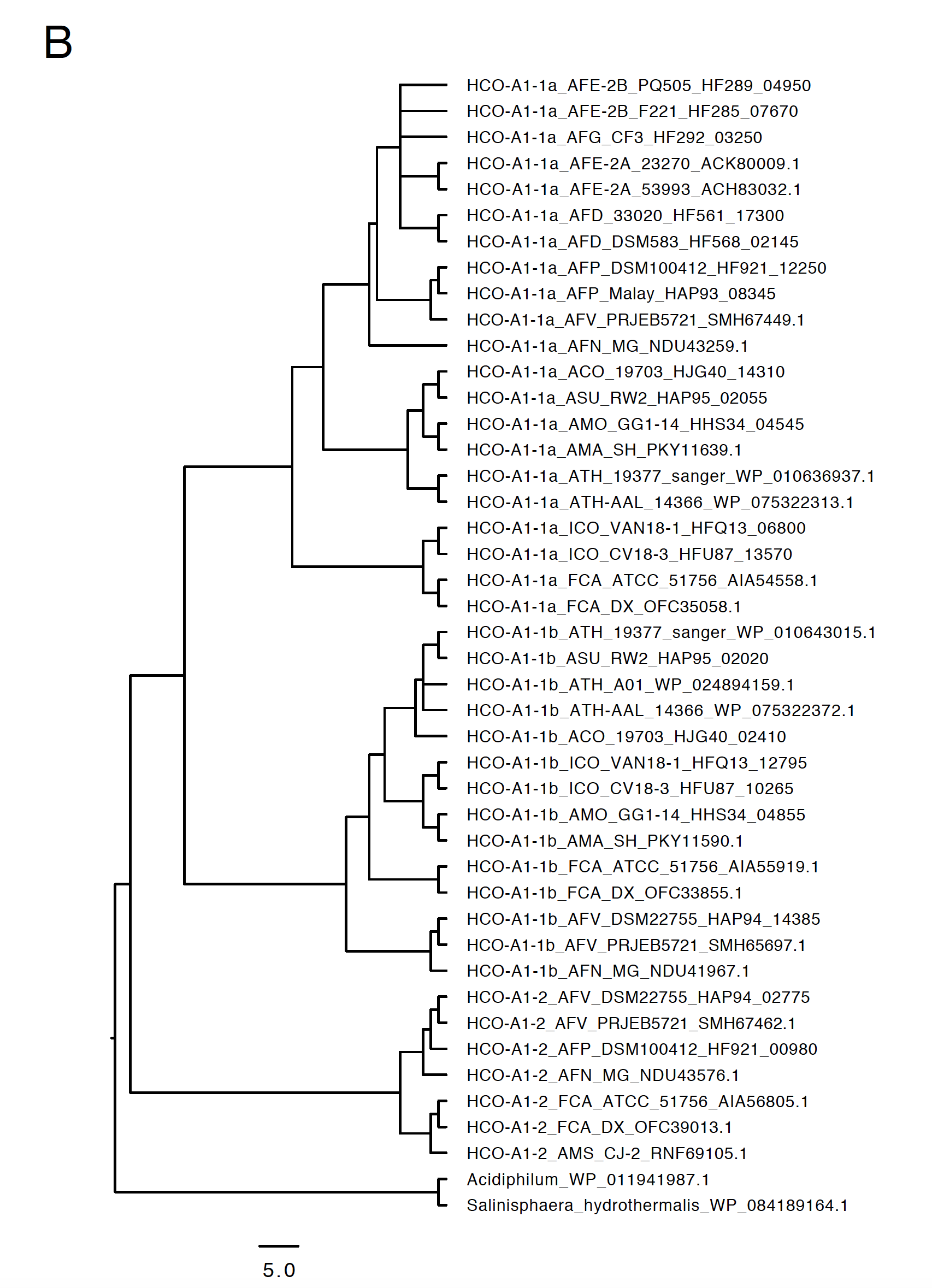
**

**Extended Figure Legends**

**Figure 4.** Metabolic traits of the *Acidithobacillia* class species. (**A**) Phyletic patterns of relevant energy metabolism genes (and associated gene clusters). Colour coding is as labelled in the figure panel. Size of the circles represents the gene dose of representative genes in each cluster. PF variants identified are detailed in Table S7c. The sulfide quinone oxidoreductase SQR, a sulfur dioxygenase SDO, the heterodisufide reductase Hdr complex (*rhd-tusA-hdrC1B1A1-orf2-hdrC2B2-gcvH-dsrE-gcvH*), the membrane-bound tetrathionate-forming thiosulfate:quinone oxidoreductase TQO (DoxDA) and the tetrathionate hydrolase TTH (TetH) are core sulfur oxidation functions. Variants of the SOX complex are lineage specific. While *T. tepidarius* and `*Am. sulfuriphilus´*, the two most ancestral lineages of the class, possess a single *sox* operon (*sox*-II: *soxXYZA-DUF302-soxB*), `*Igneacidithiobacillus´*, *`Fervidacidithiobacillus´* and sulfur-oxidizing *Acidithiobacillus,* all possessed two *sox* gene clusters (*sox*-I: *soxYZB-resB-soxXA-resC*; sox-II: *soxYZ-hyp-soxB*) pertaining to well differentiated SoxB clades (Table S7c). Partial conservation of *sox* gene cluster elements (*sox*-II: *soxYZ-hyp-soxB*) was also observed in the iron/sulfur-oxidizing early diverging species *At. ferrianus* *At. ferriphillus* and *At. ferrivorans*. However, lack of the *soxXA* cytochromes in these bacteria (and complete absence of the *sox*-I and *sox*-II elements in late-diverging iron/sulfur-oxidizers) may indicate gradual degeneration of the *sox* system in this clade of acidithiobacilli. This seems to be case also in `*Igneacidithiobacillus´* species. Despite having two copies of each of the *sox* components, operon configurations and key functional motifs (e.g. of *SoxY**, which lacks the characteristic GGCGG thiosulfate-binding motif) are not fully conserved in these species, suggesting that only one operon (*sox*-II) is functional in TS oxidation, while other is either diverging functionally or degenerating. Other PFs, such as an anaerobic molybdopterin-dependent oxidoreductase complex (DmsABC) and a sulfur oxygenase reductase (SOR), follow closely the phyletic pattern of the *sox-*II cluster. Iron oxidation PFs (*cyc2*, *rus*, *pet*-I, *pet*-II) are restricted to iron/sulfur oxidizers. The nitrogenase and Q-reducing hydrogenase (group 1) occur in iron/sulfur oxidizers and deep branching `*Am. sulfuriphilus´*, supporting the ancestral character of these traits. (**B**) Functional association networks based on gene vicinity frequencies (edges), including variants per protein family (nodes) as detailed in Table S7c. Colouring as indicated in the figure panel C. Concurrence frequency (%) of each gene pair was scored for all genomes from gene annotation tables, at selected relevant genetic contexts. Statistical concurrence information was used to colour the edges as indicated in the label and were used to derive functional modules. For example, network analysis of gene context linked genes encoding the HDR complex (in blue) with several Sox and ancillary proteins encoding genes (such as *sqr*, *sdo*, *tsd*, *doxDA* and *tetH*), pointing out to a key role of the HDR-complex during reduced inorganic sulfur compounds oxidation in the *Acidithiobacillia* class.
